# Rad52 competes with Ku70/Ku86 for binding to S-region DSB ends to modulate antibody class-switch DNA recombination

**DOI:** 10.1038/ncomms14244

**Published:** 2017-02-08

**Authors:** Hong Zan, Connie Tat, Zhifang Qiu, Julia R. Taylor, Justin A. Guerrero, Tian Shen, Paolo Casali

**Affiliations:** 1 Department of Microbiology, Immunology and Molecular Genetics, University of Texas School of Medicine, UT Health Science Center, San Antonio, Texas 78229, USA

## Abstract

Antibody class-switch DNA recombination (CSR) is initiated by AID-introduced DSBs in the switch (S) regions targeted for recombination, as effected by Ku70/Ku86-mediated NHEJ. Ku-deficient B cells, however, undergo (reduced) CSR through an alternative(A)-NHEJ pathway, which introduces microhomologies in S–S junctions. As microhomology-mediated end-joining requires annealing of single-strand DNA ends, we addressed the contribution of single-strand annealing factors HR Rad52 and translesion DNA polymerase θ to CSR. Compared with their *Rad52*^*+/+*^ counterparts, which display normal CSR, *Rad52*^*−/−*^ B cells show increased CSR, fewer intra-Sμ region recombinations, no/minimal microhomologies in S–S junctions, decreased *c-Myc/IgH* translocations and increased Ku70/Ku86 recruitment to S-region DSB ends. Rad52 competes with Ku70/Ku86 for binding to S-region DSB ends. It also facilitates a Ku-independent DSB repair, which favours intra-S region recombination and mediates, particularly in Ku absence, inter-S–S recombination, as emphasized by the significantly greater CSR reduction in *Rad52*^*−/−*^ versus *Rad52*^*+/+*^ B cells on Ku86 knockdown.

## Abstract (web summary)

Class switch DNA recombination (CSR) is critical for maturation of antibody response, and relies on Ku-mediated NHEJ of DSBs in the IgH S regions for recombination. This study shows Rad52 contributes to CSR through a Ku-independent alternative NHEJ that introduces microhomologies in S–S junctions.

Immunoglobulin (Ig) class-switch DNA recombination (CSR) and somatic hypermutation (SHM) are central to the maturation of the antibody response^1^,^2^,^3^,^4^. CSR endows antibodies with new biological effector functions by exchanging the gene encoding the Ig heavy chain constant region (C_H_) with a downstream C_H_ region. By introducing mainly point mutations in Ig V(D)J sequences, SHM provides the structural substrate for antigen-mediated selection of higher-affinity antibody mutants^1^,^2^,^4^. Similar to SHM, CSR requires activation-induced cytidine deaminase (AID)-mediated generation of DNA lesions^1^,^2^,^4^. AID, expressed in activated B cells, deaminates deoxycytosines to yield deoxyuridine:deoxyguanine mispairs^2^,^5^. These mispairs trigger DNA repair processes that lead to insertion of double-strand DNA breaks (DSBs) in the upstream (donor) and downstream (acceptor) switch (S) regions (CSR)^2^,^6^. Synapse of a S region, such as Sμ, DSB ends with DSB ends of a downstream S region, such as Sγ1, leads to deletion of the intervening DNA which is released as extrachromosomal S circle, and juxtaposition of a V_H_DJ_H_ exon to a downstream C_H_ exon cluster (in the above case Cγ1), thereby completing the CSR process^2^,^4^. Other outcomes can occur. Multiple DSBs are introduced into each of the S regions that will be the targets of recombination—Sμ being particularly prone to accumulating many DSBs. DSBs in a given S region can synapse with DSBs within the same S region, thereby yielding intra-S region deletions and non-CSR events. S-region DSB ends can also recombine with the DSB ends in other chromosomes to yield translocations, including *c-Myc/IgH* translocations^7^.

Synapsis of DSBs is generally effected by two major DNA repair pathways: non-homologous end-joining (NHEJ) or homologous recombination (HR). Unlike HR, which involves substantial DSB resection yielding overhangs with extensive sequence complementarity^8^,^9^, NHEJ synapses DSBs which have blunt/virtually blunt ends^10^,^11^. NHEJ entails recruitment of Ku70/Ku86 heterodimer, which, after binding to DSB ends, activates DNA-dependent protein kinase DNA-PKcs. This recruits the XRCC4/XRCC4-like factor/Ligase IV (Lig4) complex to complete the end-joining process^10^,^12^,^13^. Ku70/Ku86-mediated NHEJ plays an important role in recombining an upstream with a downstream S region, leading to CSR^2^,^12^,^14^,^15^. Substantial CSR, however, occurs in the absence of critical NHEJ components (Ku70/Ku86, XRCC4 or Lig4), suggesting that a Ku-independent or alternative NHEJ (A-NHEJ) DSB synapse also plays a role in CSR^12^,^16^,^17^,^18^. Although the nature of such a Ku-independent process remains to be defined, the extensive microhomologies at S–S junctions^12^,^16^,^17^,^18^,^19^ indicate that the CSR A-NHEJ involves microhomology-mediated end-joining (MMEJ). Microhomology-mediated A-NHEJ depends on moderate resection of DSBs and annealing of complementary single-strand DNA overhangs^9^,^20^,^21^. It provides the junctional mechanism effecting inter-chromosomal translocations^12^,^22^ and can be repressed by the NHEJ machinery (Ku70/Ku86, Lig4 or XRCC4)^23^,^24^,^25^,^26^,^27^.

In CSR and possibly in *c-Myc/IgH* inter-chromosomal translocations, A-NHEJ is initiated by the DNA damage sensor Parp1 and an early HR element, the DSB end-processing factor CtIP, which facilitates DSB resection to generate protruding (‘staggered') ends^28^,^29^. These are annealed through stretches of complementarity^21^,^27^, which leads to introduction of microhomologies at S–S (and *c-Myc-IgH*) junctions^16^,^18^,^29^. We contend here that microhomology-mediated A-NHEJ in CSR and *c-Myc/IgH* translocations also critically relies on another HR factor Rad52, a DNA-binding element that promotes annealing of complementary DSB single-strand ends^8^,^30^,^31^. Rad52 plays a central role in HR DSB repair and is also involved in HR-independent DSB repair^32^. We previously showed that Rad52 is recruited together with Rad51, another HR factor, to AID-resected DSB protruding ends (Rad51 recruitment to DNA DSBs is dependent on Rad52) in the human *IgH* locus during antibody diversification^33^. In addition to Rad52, the translesion DNA polymerase θ (Polθ), which promotes annealing of complementary single DNA strands, may also be involved in Ku-independent CSR. Polθ facilitates MMEJ^34^,^35^,^36^. It bypasses lesions by inserting and extending past mispairs. Polθ also copies an undamaged DNA template efficiently but in an error-prone manner and, as we have shown, plays a significant role in *Ig* locus SHM^37^. Finally, Polθ can mediate DSB synapses in chromosomal translocation and is essential for cell survival when HR is impaired^35^.

Here we addressed the role of single-strand DNA annealing factors Rad52 and Polθ in CSR. Using *Rad52*^*−/−*^ and *Polθ*^*−/−*^ B cells *in vitro* together with molecular genetic methods, we found that Rad52 deficiency profoundly altered CSR to all Ig classes, whereas Polθ deficiency did not. We validated the *in vitro* findings by analysing specific class-switched antibody responses in *Rad52*^*−/−*^ and *Polθ*^*−/−*^ mice. We adapted chromatin immunoprecipitation (ChIP) and competition assays involving recombinant proteins, to analyse the recruitment of Rad52 (RAD52) and Ku70/Ku86 (KU70/KU86) to CSR-targeted S-region DSB ends. We studied the expression kinetics of *Rad52* (*RAD52*) and *Polθ* (*POLθ*), and compared them with that of *Ku70/Ku*86 (*KU70/KU86*) in mouse and human B cells on exposure to *Aicda* (*AICDA*)/CSR-inducing stimuli. Further, we determined the impact of Rad52 deficiency on *c-Myc/IgH* translocations associated with CSR in *p53*^*−/−*^ B cells—the p53 tumour suppressor is essential for protecting B cells from *c-Myc*/*IgH* translocations and p53 deficiency significantly enhances AID-dependent *c-Myc/IgH* translocations without detectable effect on CSR^38^. Finally, we enforced Rad52 expression in normal B cells and knocked down Ku86 by specific *Ku86* short hairpin RNA (shRNA) in normal and *Rad52*^*−/−*^ B cells, to assess the reciprocal contribution of Rad52 and Ku proteins to CSR. Our findings show that Rad52 competes with Ku70/Ku86 for binding to S-region DSB-free ends to modulate CSR by facilitating a microhomology-mediated S-region DSB A-NHEJ synaptic process. This favours intra-S region recombination, but also mediates Ku-independent inter-S–S region DSB recombination.

## Results

### *Rad52*^−/−^ B cells increase CSR

**Figure 1:**
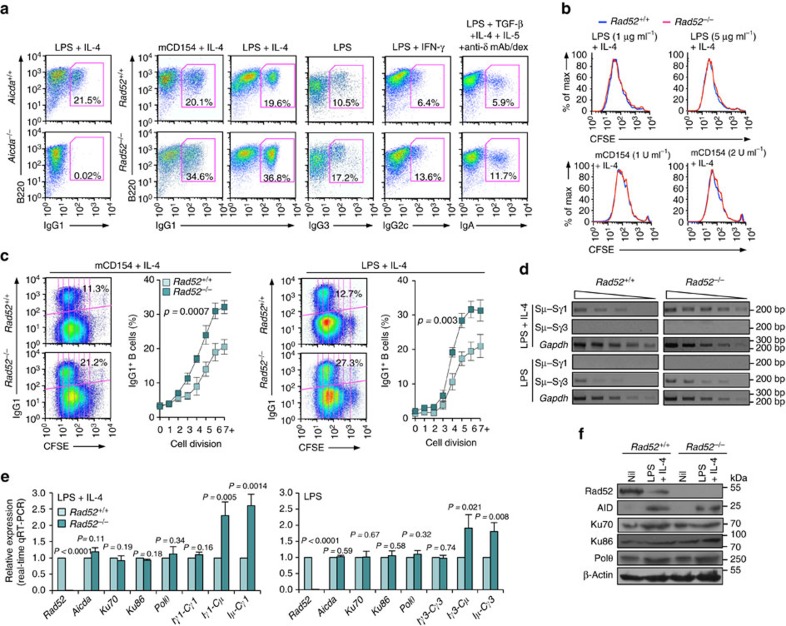
Rad52 deficiency increases CSR. (**a**) B cells purified from *Rad52*^*+/+*^ and *Rad52*^*−/−*^ C57BL/6 littermates were stimulated with mCD154 or LPS plus IL-4 (for CSR to IgG1), LPS alone (IgG3), LPS plus IFN-γ (IgG2c) or LPS plus TGF-β, IL-4, IL-5 and anti-δ mAb/dex (IgA). Purified *Aicda*^*+/+*^ and *Aicda*^*−/−*^ B cells were stimulated with LPS plus IL-4. After 96 h of culture, the cells were analysed for surface IgG1, IgG3, IgG2c or IgA by flow cytometry. (**b**) Proliferation of *Rad52*^*+/+*^ and *Rad52*^*−/−*^ B cells labelled with CFSE and stimulated for 72 h with different amounts of LPS or mCD154 plus IL-4. Progressive left shift of fluorescence intensity indicates B220^+^ B cell division. (**c**) Proliferation of *Rad52*^*+/+*^ and *Rad52*^*−/−*^ B cells labelled with the cell division tracking fluorochrome CFSE and stimulated by mCD154 plus IL-4 or LPS plus IL-4 for 96 h. CFSE intensity and surface IgG1 expression analysed by flow cytometry. Proportion of surface IgG1^+^ B cells at each cell division indicated. *P*-values determined using a paired Student's *t*-test. Data are from one representative (left panels of each condition) or mean±s.d. of three independent experiments (right panels of each condition). (**d**) Recombinant Sμ–Sγ1 or Sμ–Sγ3 DNAs analysed by digestion–circularization PCR (DC-PCR) using serially twofold diluted HindIII digested and T4 DNA ligase-ligated genomic DNA from *Rad52*^*+/+*^ or *Rad52*^*−/−*^ B cells after stimulation with LPS or LPS plus IL-4 for 96 h. *Gapdh* was used as a control for ligation and DNA loading. Data are from one representative of three independent experiments. (**e**) *Rad52*^*+/+*^ and *Rad52*^*−/−*^ B cells were cultured with LPS or LPS plus IL-4 for 60 h. *Aicda*, *Rad52*, *Ku70*, *Ku86*, *Polθ*, germline Iγ1-Cγ1 and Iγ3-Cγ3, circle Iγ1-Cμ and Iγ3-Cμ, and post-recombination Iμ-Cγ1 and Iμ-Cγ3 transcripts analysed by real-time qRT–PCR. Each sample was run in triplicate; expression normalized to *Cd79b* expression and depicted as relative to the expression in *Rad52*^*+/+*^ B cells, set as 1. Data are from three independent experiments involving three pairs of *Rad52*^*+/+*^ and *Rad52*^*−/−*^ mice (mean±s.d.). *P* values determined using a paired Student's *t*-test. (**f**) Expression of AID, Rad52, Ku70, Ku86, Polθ and β-Actin proteins in unstimulated *Rad52*^*+/+*^ and *Rad52*^*−/−*^ B cells or *Rad52*^*+/+*^ and *Rad52*^*−/−*^ B cells stimulated with LPS plus IL-4 for 72 h were analysed by immunoblotting. Data are from one representative of three independent experiments.

To test the hypothesis that Rad52, a DNA single-strand annealing (SSA) and HR factor, plays a role in CSR, we analysed B cells from *Rad52*^−/−^ C57BL/6 mice and *Rad52*^*+/+*^ C57BL/6 littermates for their ability to undergo CSR. Previous report on targeted inactivation of *Rad52* showed that Rad52 knockout reduced HR and stated that ‘Rad52 is not necessary for CSR^39^. Data, however, were not presented to support the latter claim, which was putatively based on Sμ-Sɛ recombination only. We stimulated *Rad52*^−/−^ and *Rad52*^*+/+*^ B cells, as well as *Aicda*^*−/−*^ and *Aicda*^*+/+*^ B cells with lipopolysaccharide (LPS; to induce CSR to IgG3), mCD154 or LPS plus interleukin (IL)-4 (IgG1), LPS plus interferon (IFN)-γ (IgG2c) and LPS plus transforming growth factor (TGF)-β, IL-5, IL-4 and anti-δ monoclonal antibody/dex (IgA). After 96 h of culture, the proportion of surface IgG1^+^, IgG3^+^, IgG2c^+^ and IgA^+^ B cells among *Rad52*^−/−^ B cells was ∼1.9-, 1.6-, 2.1- and 2.0-folds of those among *Rad52*^*+/+*^ B cells (Fig. 1a). This reflected an effective increase of CSR in *Rad52*^*−/−*^ B cells, as these completed the same number of divisions as their *Rad52*^+/+^ counterparts in response to different doses of LPS or mCD154 plus IL-4, while yielding a >50% increase in switched IgG1^+^ B cells (Fig. 1b,c). Increased *Rad52*^*−/−*^ B cell CSR was confirmed by detection of recombinant Sμ–Sγ1 or Sμ–Sγ3 DNA (semi-quantitative digestion–circularization PCR; Fig. 1d). Increased *Rad52*^*−/−*^ B-cell CSR was not associated with changes in germline intervening-constant heavy chain region (I_H_–C_H_) transcripts or AID, Ku70/Ku86 and Polθ levels, as shown by unchanged Iγ1-Cγ1, Iγ3-Cγ3, *Aicda*, *Ku70/Ku86* and *Polθ* transcripts (real-time quantitative reverse transcriptase–PCR (qRT–PCR)) in *Rad52*^*−/−*^ B cells, after a 60 h culture with LPS plus IL-4 or LPS alone (Fig. 1e), as well as unchanged AID, Ku70/Ku86 and Polθ protein levels (immunoblotting) after a 72 h culture with LPS plus IL-4 (Fig. 1f). This contrasted with the increased circle Iγ1-Cμ and Iγ3-Cμ transcripts, and post-recombination Iμ-Cγ1 and Iμ-Cγ3 transcripts, which are generated only after completion of CSR. Thus, intrinsic B-cell Rad52 deficiency increases CSR in the presence of normal levels of germline I_H_–C_H_ transcripts and *Aicda*, *Ku70/Ku86* and *Polθ* expression, as well as normal cell division rates.

### *Rad52*^−/−^ B cells reduce S–S junctional microhomologies

**Figure 2:**
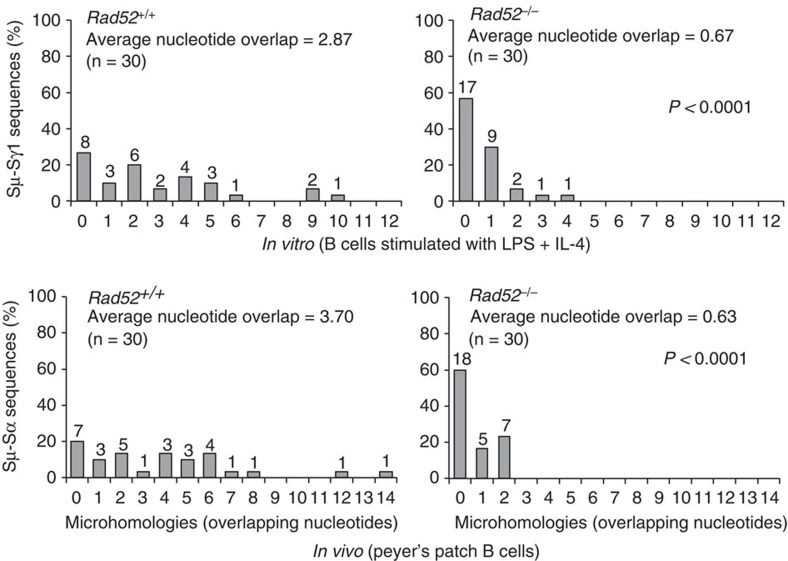
Rad52 deficiency reduces microhomologies at recombination S–S region junctions *in vitro* and *in vivo*. Histograms depict percentages of Sμ–Sγ1 junction sequences with indicated numbers of nucleotide overlaps in *Rad52*^*+/+*^ (*n*=30) and *Rad52*^*−/−*^ B cells (*n*=30) stimulated with LPS plus IL-4 for 96 h (as in Supplementary Fig. 1), and Sμ–Sα junction sequences with indicated numbers of nucleotide overlaps (microhomologies) in *Rad52*^*+/+*^ (*n*=30) and *Rad52*^*−/−*^ B cells (*n*=30) from the Peyer's patches of three pairs of *Rad52*^*+/+*^ and *Rad52*^*−/−*^ C57BL/6 littermates (as in Supplementary Fig. 2). 0 indicates no microhomology. The average length of nucleotide overlap and the numbers of sequences analysed (*n*) are indicated. *P*-values determined using a paired Student's *t*-test.

Through NHEJ, which synapses blunt or virtually blunt DSB ends, B cells complete CSR, leading to S–S junctions with no or minimal overlapping sequences (microhomologies)^12^. In B cells deficient in Ku70/Ku86 or other key NHEJ factors, such as XRCC4 and Lig4, CSR is reduced but not abolished, with the residual switched B cells displaying increased frequency and length of microhomologies at S–S junctions^12^,^16^,^17^,^18^,^19^. As we hypothesized, such S–S junctional microhomologies would be introduced by annealing of DSB single-strand DNA overhangs, a function characteristic of Rad52 (refs 23, 27). If our hypothesis were correct, then the increased CSR in *Rad52*^−/−^ B cells should result in decreased frequency and length of S–S junctional microhomologies. We analysed Sμ–Sγ1 junctions from *Rad52*^−/−^ and *Rad52*^+/+^ B cells stimulated with LPS plus IL-4 for 96 h, as well as Sμ–Sα junctions *ex vivo* from B220^+^PNA^hi^ germinal centre (GC) B cells in Peyer's patches. As expected, the majority of Sμ–Sγ1 and Sμ–Sα junctions in *Rad52*^+/+^ B cells displayed microhomologies, with >73% of Sμ–Sγ1 and 77% of Sμ–Sα junctional microhomologies consisting of up to 14 nucleotides, and >36% of Sμ–Sγ1 and 46% of Sμ–Sα junctions with microhomologies of 4 nucleotides or more. The frequency and length of Sμ–Sγ1 and Sμ–Sα junctional microhomologies were significantly reduced in *Rad52*^*−/−*^ B cells (Supplementary Figs 1 and 2, and Fig. 2). In these B cells, 57% of Sμ–Sγ1 and 60% of Sμ–Sα junctions contained no microhomology, and only 3.3% of Sμ–Sγ1 and none of Sμ–Sα junctions contained microhomologies of 4 nucleotides or more. Overall, the average microhomologous sequence decreased from 2.87 (±0.66) nucleotides (Sμ–Sγ1) and 3.70 (±0.74) nucleotides (Sμ–Sα) in *Rad52*^*+/+*^ B cells to 0.67 (±0.17) (Sμ–Sγ1, *P<*0.0001, paired Student's *t*-test) and 0.63 (±0.21) (Sμ–Sα, *P<*0.0001, paired Student's *t*-test) nucleotide in *Rad52*^*−/−*^ B cells. The dramatic increase in S–S junctions without microhomologies and reduced length of S–S junctional microhomologies in *Rad52*^*−/−*^ B cells suggested that the greater number of CSR events in these B cells reflected the activity of NHEJ, which relies on Ku70/Ku86 and introduces no or minimal microhomologies.

### *Polθ*^−/−^ B cells display normal CSR and S–S microhomologies

**Figure 3:**
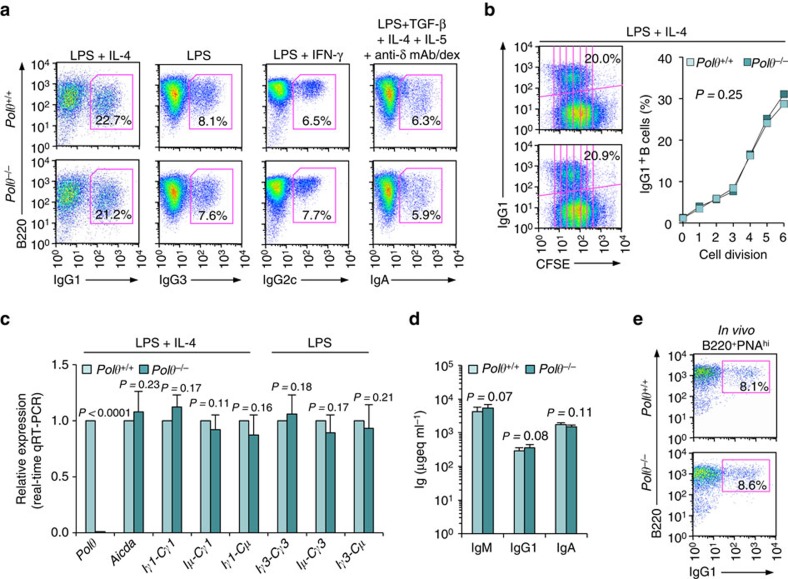
*Polθ*^*−/−*^ B cells undergo normal CSR *in vitro* and *in vivo*. (**a**) *Polθ*^*+/+*^ and *Polθ*^*−/−*^ B cells were stimulated with LPS plus IL-4 (for CSR to IgG1), LPS alone (IgG3), LPS plus IFN-γ (IgG2c) or LPS plus TGF-β, IL-4, IL-5 and anti-δ mAb/dex (IgA). After a 96 h of culture, the B cells were analysed for surface IgG1, IgG3, IgG2c or IgA by flow cytometry. (**b**) Proliferation of *Polθ*^*+/+*^ and *Polθ*^*−/−*^ B cells labelled with CFSE and stimulated by LPS plus IL-4 for 96 h. CFSE intensity and surface IgG1 expression analysed by flow cytometry. Proportion of surface IgG1^+^ B cells at each cell division indicated. *P*-values determined using a paired Student's *t*-test. Data are from one representative of three independent experiments. (**c**) *Polθ*^*+/+*^ and *Polθ*^*−/−*^ B cells cultured with LPS or LPS plus IL-4 for 60 h. Expression of *Polθ*, *Aicda*, germline Iγ1-Cγ1 and Iγ3-Cγ3, circle Iγ1-Cμ and Iγ3-Cμ, and post-recombination Iμ-Cγ1 and Iμ-Cγ3 transcripts analysed by qRT–PCR and normalized to *Gapdh* transcript, depicted as relative to the expression of each transcript in *Polθ*^*+/+*^ B cells, set as 1. Each sample was run in triplicate. Data are from three independent experiments involving three pairs of *Polθ*^*+/+*^ and *Polθ*^*−/−*^ mice (mean±s.d.). *P*-values determined using a paired Student's *t*-test. (**d**,**e**) *Polθ*^*+/+*^ and *Polθ*^*−/−*^ littermates were injected with NP_16_-CGG and killed 10 days later. (**d**) Titres of circulating IgM, IgG1 and IgA analysed by enzyme-linked immunosorbent assay (ELISA), expressed as μgeq ml^−1^. *P*-values determined using a paired Student's *t*-test. Data are from three independent experiments (mean±s.d.). (**e**) Surface IgG1 expression in spleen B220^+^PNA^hi^ GC B cells analysed by flow cytometry. Data are from one representative of three independent experiments.

The error-prone translesion DNA Polθ, which, as we have shown, plays a role in SHM^37^, can anneal and end-join DNA single-strand ends, leading to the introduction of microhomologies^34^,^35^,^36^, as in the CSR S–S region synaptic process^40^. To determine whether Polθ is actually involved in CSR, we stimulated *Polθ*^−/−^ and *Polθ*^*+/+*^ B cells with LPS plus IL-4, LPS only, LPS plus IFN-γ and LPS plus TGF-β, IL-5, IL-4 and anti-δ monoclonal antibody/dex, to induce CSR to IgG1, IgG3, IgG2c and IgA, respectively. After 96 h of culture, the proportions of class-switched IgG1^+^, IgG3^+^, IgG2c^+^ and IgA^+^ *Polθ*^−/−^ B cells were comparable to those of *Polθ*^*+/+*^ B cells, as were B-cell divisions and the proportion of class-switched cells/round of cell division (Fig. 3a,b). The normal CSR in *Polθ*^−/−^ B cells was supported by the normal levels of *Aicda* expression, circle Iγ1-Cμ and Iγ3-Cμ transcripts, post-recombination Iμ-Cγ1 and Iμ-Cγ3 transcripts, as well as germline Iγ1-Cγ1 and Iγ3-Cγ3 transcripts (that is, comparable to *Polθ*^*+/+*^ B cells) (Fig. 3c). In addition, *Polθ*^−/−^ B cells were comparable to *Polθ*^*+/+*^ B cells in Sμ–Sγ1 junction frequency of microhomologies and length (*P=*0.09, paired Student's *t*-test) (Supplementary Fig. 3). Finally, that *Polθ*^−/−^ B cells functioned normally in CSR was confirmed by analyses *in vivo* showing comparable levels of serum IgM, IgG1 and IgA, as well as IgG1^+^ B220^+^PNA^hi^ GC B cells in the spleens from *Polθ*^−/−^ and *Polθ*^*+/+*^ littermates that had been injected with NP_16_-CGG (Fig. 3d,e). Thus, the DNA single-strand annealing Polθ does not apparently play a role in CSR synapsing of S–S region DSBs.

### *Rad52* deficiency increases class-switched antibody responses

**Figure 4:**
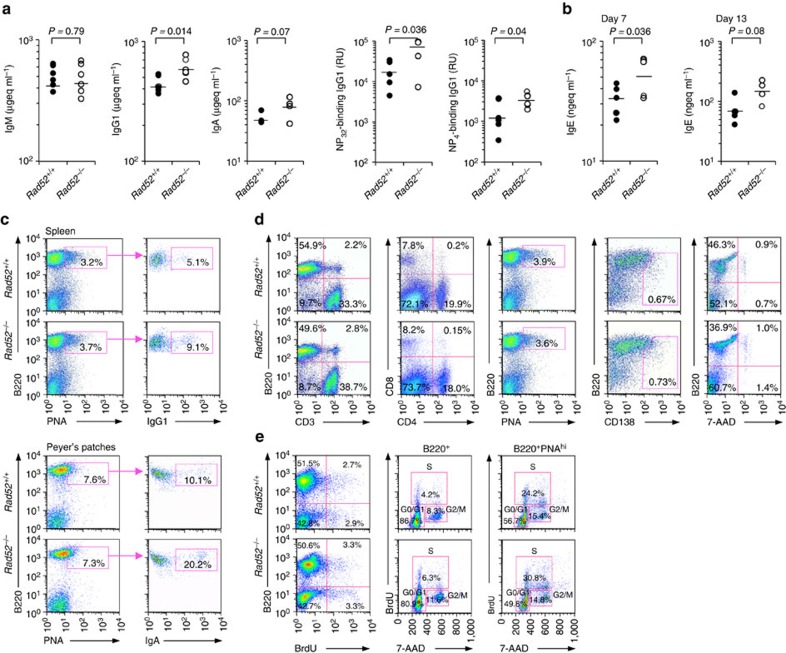
Rad52 deficiency increases class-switching in the antibody response *in vivo.* (**a**) *Rad52*^*+/+*^ and *Rad52*^*−/−*^ littermates were injected with NP_16_-CGG and killed 10 days later. Titres of circulating total IgM, IgG1 and IgA, NP_32_-binding and (high affinity) NP_4_-binding IgG1 analysed by enzyme-linked immunosorbent assay (ELISA), expressed as μgeq ml^−1^ or number of dilutions needed to reach 50% of saturation binding (relative units, RU). (**b**) *Rad52*^*+/+*^ and *Rad52*^*−/−*^ littermates were injected twice with OVA at day 0 and day 7. Titres of circulating total IgE analysed by ELISA at day 7 (before the second OVA injection) and day 13, expressed as ngeq ml^−1^. Each symbol represents an individual mouse, *n=*5 or 6 pairs of mice. *P*-values determined using a paired Student's *t*-test. (**c**) Surface IgG1 expression in spleen B220^+^PNA^hi^ GC B cells or IgA expression in Peyer's patch B220^+^PNA^hi^ GC B cells of NP_16_-CGG-injected *Rad52*^*+/+*^ and *Rad52*^*−/−*^ littermates analysed by flow cytometry. (**d**) Flow cytometry analysis of spleen cells from NP_16_-CGG-injected *Rad52*^*+/+*^ and *Rad52*^*−/−*^ littermates for the proportion of: B220^+^ B cells and CD3^+^ T cells, CD4^+^ and CD8^+^ T cells, B220^+^PNA^hi^ GC B cells, B220^lo^CD138^+^ plasma cells and viable (7-AAD^*–*^) B220^+^ B cells. (**e**) *Rad52*^*+/+*^ and *Rad52*^*−/−*^ littermates were injected with NP_16_-CGG. Ten days after the injection, the mice were injected i.p. with BrdU twice within a 16 h interval and killed 4 h after the last injection. Left panels: proliferating B cells (BrdU-stained B220^+^ B cells) analysed by flow cytometry; middle and right panels: *in vivo* cell cycle analysis (quadrant corresponding to the G0/G1, S and G2/M phase of the cell cycle) of B220^+^ B cells and B220^+^PNA^hi^ GC B cells. Data are from one representative of three independent experiments.

To define the impact of Rad52 deficiency on CSR *in vivo*, we analysed the class-switched antibody response to 4-Hydroxy-3-nitrophenyl acetyl hapten (NP) in *Rad52*^−/−^ mice and *Rad52*^*+/+*^ littermates after injection of NP-conjugated chicken gamma globulin (NP_16_-CGG), which preferentially induces T-dependent NP-specific IgG1 antibodies. *Rad52*^−/−^ mice showed significantly higher titres of NP_32_-binding IgG1 and (high affinity) NP_4_-binding IgG1, as well as total IgG1 and IgA than their *Rad52*^+/+^ counterparts, in the presence of normal IgM levels (Fig. 4a). In addition, similar *Rad52*^−/−^ mice showed significantly higher titres of total IgE than their *Rad52*^*+/+*^ littermates after injection of ovalbumin (OVA; Fig. 4b). In *Rad52*^−/−^ mice, high IgG1 and IgA, and NP-binding IgG1 titres were associated with a >75% increase of IgG1^+^ B220^+^PNA^hi^ GC B cells in the spleen (Fig. 4c) and a 100% increase in IgA^+^ B220^+^PNA^hi^ GC B cells in Peyer's patches. In *Rad52*^−/−^ mice, the spleen size, and number and size of Peyer's patches were comparable to those in their *Rad52*^+/−^ and *Rad52*^+/+^ counterparts. Consistent with a previous report^39^, the number of B (B220^+^) and T (CD3^+^) cells, and the proportions of CD4^+^ and CD8^+^ T cells in *Rad52*^−/−^ mice were comparable to those in their *Rad52*^+/+^ littermates. In addition, the proportion of B220^+^PNA^hi^ GC B cells and B220^low^ CD138^+^ plasma cells in the spleen were also comparable in *Rad52*^−/−^ and *Rad52*^+/+^ mice; *Rad52*^−/−^ mice showed normal B-cell viability (Fig. 4d), proliferation and normal cell cycle in total and GC B cells, as measured by bromodeoxyuridine (BrdU) incorporation and 7-aminoactinomycin D (7-AAD) staining (Fig. 4e). Thus, Rad52 deficiency leads to significantly increased class-switched antibody response without affecting B-cell proliferation or survival.

### Rad52 competes with Ku70/Ku86 for binding to S-region DSB ends

**Figure 5:**
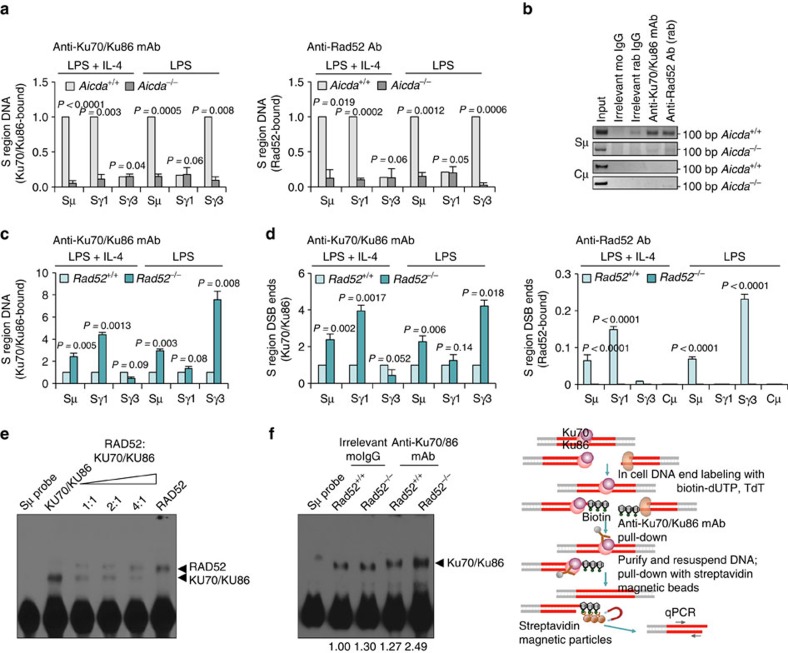
Rad52 competes with Ku70/Ku86 for binding to S-region DSB ends. (**a**) Ku70/Ku86 and Rad52 are recruited to CSR-targeted S region DNA in an AID-dependent manner in B cells undergoing CSR. *Aicda*^*+/+*^ and *Aicda*^*−/−*^ B cells were stimulated with LPS or LPS plus IL-4 for 60 h. Cross-linked chromatin was precipitated using rabbit anti-Rad52 antibody or mouse anti-Ku70/Ku86 mAb. Precipitated Sμ, Sγ1 and Sγ3 DNA quantified by real-time quantitative PCR (qPCR); amounts relative to those in *Aicda*^*+/+*^ B cells, set as 1. Each precipitated DNA sample was run as triplicate in qPCR; the average of each triplicate was used as data point for that individual sample. Data are from three independent experiments involving three pairs of *Aicda*^*+/+*^ and *Aicda*^*−/−*^ mice (mean±s.d.). *P*-values determined using a paired Student's *t*-test. (**b**) Precipitated Sμ and Cμ DNA from *Aicda*^*+/+*^ and *Aicda*^*−/−*^ B cells detected by PCR. Data are one representative of three independent experiments. (**c**) *Rad52*^*+/+*^ and *Rad52*^*−/−*^ B cells were stimulated with LPS or LPS plus IL-4 for 60 h. Chromatin was cross-linked and precipitated using a mouse anti-Ku70/Ku86 mAb. The precipitated Sμ, Sγ1 and Sγ3 DNAs quantified by real-time qPCR; amounts in *Rad52*^*−/−*^ B cells are as relative to those in *Rad52*^*+/+*^ B cells, set as 1. Each precipitated DNA sample was run as triplicate in qPCR; the average of each triplicate was used as data point for that individual sample. Data are from three independent experiments involving three pairs of *Rad52*^*+/+*^ and *Rad52*^*−/−*^ mice (mean±s.d.). *P*-values determined using a paired Student's *t*-test. (**d**) *Rad52*^*+/+*^ and *Rad52*^*−/−*^ B cells were stimulated with LPS or LPS plus IL-4 for 60 h. DNA ends were labeled *in situ* with bio-dUTP using TdT. Chromatin was cross-linked and precipitated using mouse anti-Ku70/Ku86 mAb (left panel) or rabbit anti-Rad52 antibody (right panel). After resuspension, DNA with broken ends was pulled down with streptavidin magnetic beads. Precipitated Sμ, Sγ1, Sγ3 and Cμ DNAs quantified by real-time qPCR (this approach allowed for detection of Ku70/Ku86 or Rad52 bound to DSB-free ends); amounts of precipitated DNA are relative to those in *Rad52*^*+/+*^ B cells, set as 1 (left panel), or relative to respective input DNA (right panel). Each precipitated DNA sample was run as triplicate in qPCR; the average of each triplicate was used as data point for that individual sample. Data are from three independent experiments involving three pairs of *Rad52*^*+/+*^ and *Rad52*^*−/−*^ mice (mean±s.d.). *P*-values determined using a paired Student's *t*-test. (**e**,**f**) EMSA using a biotin-labelled double-stranded Sμ DNA probe and (**e**) recombinant human RAD52 and KU70/KU86 (constant amount) proteins at different ratios (1:1, 2:1 and 4:1), or (**f**) nuclear extracts (10 μg protein) from *Rad52*^*+/+*^ and *Rad52*^*−/−*^ B cells (stimulated with LPS plus IL-4 for 60 h) incubated with mouse anti-Ku70/Ku86 mAb or irrelevant mouse IgG (control). The formation of protein–DNA complexes was shifted by anti-Ku70/Ku86 mAb. Numbers below gel image indicate relative density of protein–DNA complex bands normalized with the density of free probe. Shown is one representative gel of three independent experiments.

The increased CSR in *Rad52*^*−/−*^ B cells *in vitro* and *in vivo* suggested that Rad52 is recruited to S-region DSB-ends where it would compete with Ku70/Ku86 to modulate CSR. To demonstrate that Rad52 and Ku70/Ku86 indeed bind to CSR-targeted S regions, we performed ChIP assays with anti-Rad52 antibody and anti-Ku70/Ku86 monoclonal antibodies. These showed that similar to Ku70/Ku86, Rad52 was specifically recruited to Sμ and Sγ1 regions, not Sγ3, in B cells stimulated by LPS plus IL-4 (undergoing CSR to IgG1), and to Sμ and Sγ3, not Sγ1, in B cells stimulated by LPS (CSR to IgG3). Rad52 and Ku70/Ku86 could be readily detected on Sμ, and Sγ1 or Sγ3, but not Cμ region, in *Aicda*^*+/+*^ B cells stimulated with LPS plus IL-4 or LPS alone, but failed to associate with such S regions in similarly activated *Aicda*^*−/−*^ B cells, showing that similar to Ku70/Ku86, recruitment of Rad52 to CSR-targeted S regions was dependent on S region AID processing (Fig. 5a,b). To analyse the impact of Rad52 on Ku70/Ku86 recruitment to CSR-targeted S regions, we performed ChIP assays using anti-Ku70/Ku86 monoclonal antibody in *Rad52*^*+/+*^ and *Rad52*^*−/−*^ B cells stimulated with LPS plus IL-4 or LPS. We complemented this approach with *in situ* DNA end-labelling by biotin-16-dUTP (bio-dUTP)^41^ followed by ChIP with anti-Ku70/86 monoclonal antibody or anti-Rad52 antibody and capture of biotin-labelled DNA fragments with streptavidin magnetic beads. This approach, which allows for detection of Ku70/Ku86 or Rad52 on DSB ends, showed that Rad52 indeed bound to the free ends of DSBs in the CSR-targeted S regions, but not to Cμ region or S regions not involved in CSR. In the absence of Rad52, Ku70/Ku86 bound to CSR-targeted Sγ1 and Sγ3 DSB free ends, up to 7.6-fold more than in the presence of Rad52 (Fig. 5c,d).

To prove that Rad52 competes with Ku70/Ku86 for binding to S-region DSB ends, we incubated recombinant human RAD52 and/or KU70/KU86 proteins with an 84 bp biotin-labelled double-strand Sμ probe and then submitted the reactants to electrophoretic mobility shift assays (EMSAs). Incubation of KU70/KU86 or RAD52 with biotin-Sμ DNA probe alone gave rise to a KU70/KU86 or RAD52 protein–Sμ DNA complex (Fig. 5e). Incubation of increasing amounts of recombinant human RAD52 protein with the same amount of KU70/KU86 led to increasing binding of RAD52 and decreasing binding of KU70/KU86 to biotin-Sμ DNA probe, indicating that RAD52 competed with KU70/KU86 for binding to Sμ DSB ends. Further, incubation of cell extracts from *Rad52*^*+/+*^ B cells stimulated with LPS plus IL-4 with the biotin-Sμ DNA probe gave rise to protein–DNA complexes containing Ku70/Ku86, as determined by supershift with an anti-Ku70/Ku86 monoclonal antibody (Fig. 5f). Such DNA–Ku70/Ku86 complexes were increased by about twofold in *Rad52*^*−/−*^ B cells—relative density of the protein–DNA complex bands changed from 1.27 to 2.49. Thus, Rad52 competes with Ku70/Ku86 for binding to CSR-targeted S-region DSB ends.

### *Rad52*^*−/−*^ B cells reduce *c-Myc/IgH* translocations

**Figure 6:**
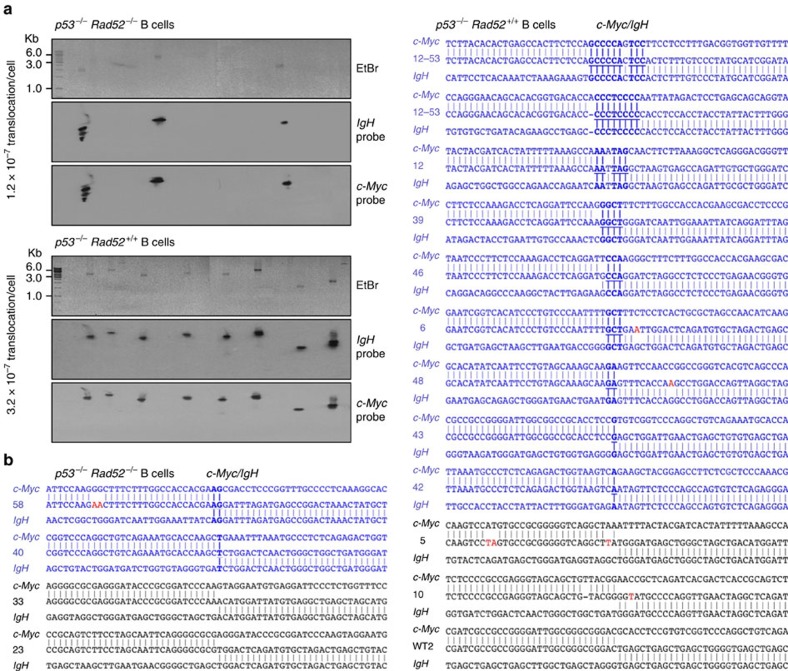
Rad52 deficiency reduces *c-Myc/IgH* translocations and microhomologies at *c-Myc–IgH* junctions. B cells from *p53*^*−/−*^*Rad52*^*+/+*^ and *p53*^*−/−*^*Rad52*^*−/−*^ mice were stimulated with LPS plus IL-4 for 96 h before genomic DNA isolation. (**a**) *c-Myc/IgH* translocations were identified by amplifying DNA using long-range nested PCR involving primers specific to the *IgH* and *c-Myc* locus, and verified by Southern blot hybridization with an *IgH* or *c-Myc-*specific probe. Each PCR assay was performed using template DNA from 10^6^ cells. Twenty-five amplicons from *p53*^*−/−*^*Rad52*^*+/+*^ B cells and 25 amplicons from *p53*^*−/−*^*Rad52*^*−/−*^ B cells are shown. The frequencies of *c-Myc/IgH* translocations per cell are indicated below the gel images. PCR amplification products that can be detected by both *IgH* and *c-Myc* DNA probes were from *c-Myc/IgH* translocations: 8 of 25 and 3 of 25 amplicons from *p53*^*−/−*^*Rad52*^*+/+*^ and *p53*^*−/−*^*Rad52*^*−/−*^ B cells, respectively, contained PCR amplification products from *c-Myc/IgH* translocations. (**b**) Sequences of the *c-Myc/IgH* translocation junctions. Amplified *c-Myc*–*IgH* junctional DNAs were cloned and sequenced. Each sequence is compared with germline *c-Myc* (above) and *IgH* (below) sequences. Microhomologies are bold and underlined. Sequences containing microhomologies are in blue. Point mutations are in red. Data are from three pairs of mice.

Not only does CSR introduce DSBs in S regions but it can also instigate translocations between S regions on mouse chromosome 12 and exon 1 of the *c-Myc* gene on chromosome 15 (ref. 12). In addition to decreasing CSR, deficiency of critical NHEJ elements, such as XRCC4, Ku70 or combined Ku70 and Lig4, results in greatly increased *c-Myc/IgH* translocations, as mediated by A-NHEJ^16^,^17^,^25^,^42^. Such translocations are characterized by microhomologies at *c-Myc-IgH* junctions, the expression of a DSB end-joining process initiated by annealing of DSB single-strand DNA overhangs. We reasoned that in *Rad52*^*−/−*^ B cells, increased CSR, a reflection of the enhanced recruitment of Ku70/Ku86 to S-region DSBs and increased NHEJ activity, would be associated with a decreased frequency of *c-Myc/IgH* translocations, possibly including only limited microhomologies, as a result of lack of Rad52 single-strand DNA annealing activity. To prove that Rad52 deficiency has a negative impact on *c-Myc/IgH* translocations, we bred *p53*^*−/−*^ mice with *Rad52*^*+/–*^ mice to generate *p53*^*−/−*^*Rad52*^*+/+*^ and *p53*^*−/−*^*Rad52*^*−/−*^ mice—p53 deficiency significantly enhances AID-dependent *c-Myc/IgH* translocations but has no detectable effect on CSR^38^. We stimulated *p53*^*−/−*^*Rad52*^*+/+*^ and *p53*^*−/−*^*Rad52*^*−/−*^ B cells with LPS plus IL-4. After 96 h of culture, we detected *c-Myc/IgH* translocations by long-range PCR and confirmed their identity by Southern blot hybridization (using *c-Myc* and *IgH* probes) and sequencing. In *p53*^*−/−*^*Rad52*^*−/−*^ B cells, *c-Myc/IgH* translocations were induced at a frequency (1.2 × 10^−7^ translocation per cell) about threefold lower than in *p53*^*−/−*^*Rad52*^*+/+*^ B cells (3.2 × 10^−7^ translocation per cell, *P=*0.011, paired Student's *t*-test) and comprised a lower number of microhomologies at *c-Myc*–*IgH* junctions (*P*=0.0014, paired Student's *t*-test; Fig. 6). Thus, the greatly reduced frequency of *c-Myc/IgH* translocations in *p53*^*−/−*^*Rad52*^*−/−*^ B cells undergoing CSR supports a role of Rad52 in the single-strand DNA annealing process in inter-chromosomal translocations.

### *Rad52*^*−/−*^ B cells reduce intra-S region DNA recombination

**Figure 7:**
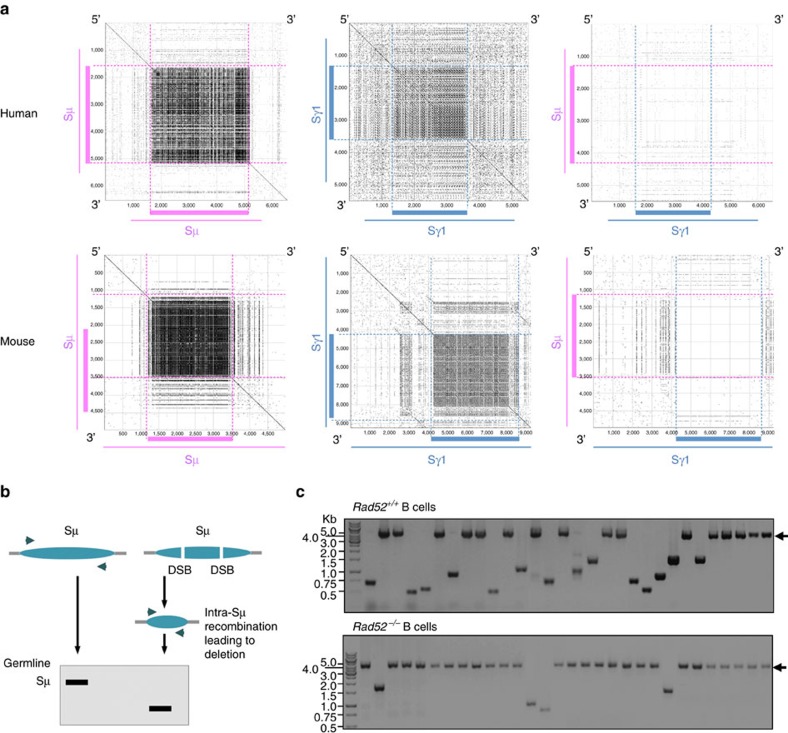
Rad52-mediated S-region DSB repair favours intra-S region DNA recombination. (**a**) Each S region consists of highly repetitive motifs, which can facilitate the formation of microhomologies, in particular within the S region core. As the characteristically repetitive sequences are virtually unique to S regions, DSB ends in the same S region are better suited substrates for Rad52-mediated complementary DNA single-strand annealing than those in two different S regions, such as Sμ and Sγ1. Repetitive sequence elements in human and mouse Sμ and Sγ1 that can potentially form microhomologies were identified by Pustell Matrix dot plot using MacVector software and are depicted by small dots. Thick lines indicate the core regions of Sμ and Sγ1. (**b**) Schematic representation of the detection of intra-S region recombination (deletion) in Sμ region by PCR amplification. DNA-amplified sequences of Sμ region that underwent intra-S region DNA recombination are shorter than those of Sμ in the germline configuration. (**c**) *Rad52*^*+/+*^ and *Rad52*^*−/−*^ B cells were stimulated with LPS plus IL-4 for 96 h. Sμ region DNA was amplified by nested PCR. PCR amplification products were then cloned into the TOPO cloning vector. Sμ region sequences from individual clones amplified by PCR and resolved through a 0.8% agarose gel. PCR amplification products smaller than that amplified from the germline Sμ region DNAs (indicated by arrows) are from Sμ region DNAs that underwent intra-S region recombination, thereby deleting variable lengths of DNA: 14 of 30 Sμ region DNAs in *Rad52*^*+/+*^ B cells and 4 out of 30 Sμ region DNAs in *Rad52*^*−/−*^ B cells underwent intra-S region recombination. Data are from three pairs of *Rad52*^*+/+*^ and *Rad52*^*−/−*^ C57BL/6 mice.

AID generates multiple DSBs within the targeted S regions, many of which are rejoined or joined to other DSBs within the same S region^12^. Each S region consists of highly repetitive DNA motifs. These can give rise to complementary protruding ends in upstream and downstream DSBs that are suitable substrates for DNA annealing by Rad52. Subsequent synapse of such DSB ends leads to intra-S region recombination and deletion of intervening DNA (of variable length). All S regions include characteristic and highly repetitive motifs. Such highly repetitive motifs differ in both nature and frequency in different S regions. It follows that DSB protruding ends from two different S regions, such as Sμ and Sγ1, will encompass sequences of virtually no complementarity, making them poor substrates for Rad52-mediated complementary annealing than protruding ends from DSBs within the same S region. Indeed, Pustell Matrix dot plot analysis of human and mouse Sμ and Sγ1 revealed maximal sequence complementarity within the individual Sμ or Sγ1 region, in particular within their core region, and maximal lack of complementarity between these two S regions, in particular in their core sequences (Fig. 7a). Accordingly, the reduced CSR in Ku70 deficiency, which, as we have hypothesized, would result in increased Rad52 recruitment to S-region DSB-ends, was shown to be associated with significantly increased occurrence of intra-S region deletions, remnants of occurred intra-S region recombinations^17^.

We hypothesize here that such increased intra-S region deletions are mediated by a DSB synaptic process involving Rad52. If our hypothesis is correct, then the absence of Rad52—which, as shown by the preceding experiments, led to increased recruitment of Ku70/Ku86 to S-region DSB ends and increased CSR—would result in reduction of intra-S region deletions. To test our hypothesis, we set up to assay for deletions within the Sμ region of genomic DNA from *Rad52*^*+/+*^ and *Rad52*^*−/−*^ B cells activated for CSR to IgG1—intra-S region deletions occur far more frequently in Sμ than the other (downstream) S regions^17^—using specific primers flanking both sides of Sμ to amplify this DNA region. Sμ DNAs with internal deletions were detected by visualizing amplification products that were shorter than germline Sμ and then positively identified by DNA sequencing (Fig. 7b,c and Supplementary Fig. 4). This revealed that *Rad52*^*−/−*^ B cells displayed a substantially lower frequency of intra-Sμ region deletions as compared with their *Rad52*^*+/+*^ counterparts (4 out of 30, that is, 13.3%, versus 14 out 30, that is, 46.7%, *P*=0.0006, paired Student's *t*-test). Even taking into account a remote possibility that some of the S-region DNA deletions might have been stemmed from PCR amplification or propagation of S region-containing plasmids in bacteria, these findings indicate that the Rad52-mediated DSB synaptic process favours intra-S region over inter-S–S region recombination.

### Rad52 and Ku86 reciprocally modulate CSR

**Figure 8:**
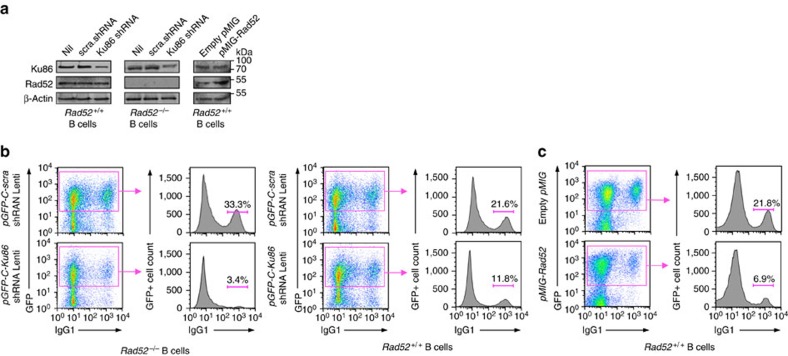
Knockdown of Ku86 expression in *Rad52*^*−/−*^ B cells virtually ablates CSR and enforced expression of Rad52 in normal B cells significantly impairs CSR. *Rad52*^*−/−*^ and *Rad52*^*+/+*^ B cells were transduced with pGFP-C-Ku86-shRNALenti lentiviral vector expressing *Ku86*-specific shRNA and GFP, or pGFP-C-scr-shRNALenti lentiviral vector expressing scrambled shRNA and GFP. *Rad52*^*+/+*^ B cells were activated with LPS for 12 h and transduced with empty pMIG-GFP retroviral vector or pMIG-GFP-Rad52 retroviral vector expressing recombinant Rad52. After lentiviral or retroviral transduction, B cells were cultured for 96 h with LPS plus IL-4. (**a**) B-cell expression of Ku86, Rad52 and β-Actin proteins analysed by immunoblotting. (**b**) Proportions of surface IgG1^+^ B cells among pGFP-C-scr-shRNALenti or pGFP-C-Ku86-shRNALenti lentiviral vector-transduced (B220^+^GFP^+^) *Rad52*^*−/−*^ and *Rad52*^*+/+*^ B cells were analysed by flow cytometry. Data are from one representative of three independent experiments. (**c**) Proportions of surface IgG1^+^ B cells among empty pMIG-GFP or pMIG-GFP-Rad52 retroviral vector-transduced (B220^+^GFP^+^) *Rad52*^*+/+*^ B cells were analysed by flow cytometry. Data are from one representative of three independent experiments.

To define whether, in addition to mediating intra-S region recombination, Rad52 can also mediate inter-S–S region recombination, we knocked down Ku86 in *Rad52*^−/−^ and *Rad52*^+/+^ B cells using a pGFP-C-Ku86-shLenti lentiviral vector. Expression of a *Ku86-*specific shRNA reduced Ku86 protein level by 87% (average) (when taking into consideration the 80% lentiviral transduction efficiency in our experiments) without altering Rad52 protein expression (Fig. 8a)—a pGFP-C-scrambled-shLenti lentiviral vector, which did not alter Ku86 expression, was used as a control. The transduced B cells were cultured with LPS and IL-4 for 96 h before analysing CSR to IgG1. Similar to untransduced *Rad52*^−/−^ B cells, pGFP-C-scrambled-shLenti lentiviral vector-transduced *Rad52*^−/−^ B cells displayed a (54%) higher level of CSR to IgG1 (*P*=0.003, paired Student's *t*-test) as compared with their *Rad52*^+/+^ counterparts (Fig. 1a,b). In similar *Rad52*^−/−^ B cells, Ku86 knockdown by pGFP-C-Ku86-shLenti lentiviral vector resulted in a nearly tenfold reduction of CSR to IgG1, as compared with the twofold reduction in *Rad52*^*+/+*^ B cells transduced by the pGFP-C-Ku86-shLenti lentiviral vector—knockdown of Ku86 in *Rad52*^*+/+*^ B cells reduced CSR to an extent comparable to that reported for Ku70- or Ku86-deficient B cells^18^. The less profound CSR reduction by Ku86 knockdown in *Rad52*^+/+^ than *Rad52*^−/−^ B cells reflected the contribution of Rad52 to inter-S–S region recombination in *Rad52*^+/+^ B cells—the residual 3.4% CSR in pGFP-C-Ku86-shLenti lentiviral vector-transduced *Rad52*^−/−^ B cells was (probably) due to the residual (∼12%) functional Ku86 in these B cells (Fig. 8a). Although knockdown of Ku86 in *Rad52*^−/−^ B cells virtually ablated CSR, knockdown of Ku86 in *Rad52*^*+/+*^ B cells resulted only in partial CSR reduction, thereby pointing at a contribution of Rad52 to inter-S–S region synapses in the absence of Ku86. Thus, Rad52 partially rescues CSR in B cells with a compromised Ku-dependent/NHEJ repair pathway.

Our experiments have shown that in the absence of Ku86, Rad52 can mediate inter-S–S region synapses. In the presence of Ku70/Ku86, Rad52 would compete with this heterodimer for binding to S-region DSB ends, thereby skewing the S-region DSB synaptic process towards intra-S region recombination to the detriment of inter-S–S region recombination and CSR. To test the hypothesis that overexpression of Rad52 will further reduce recruitment of Ku70/Ku86 to S-region DSB ends and significantly dampen CSR, we transduced *Rad52*^*+/+*^*Ku86*^*+/+*^ (normal) B cells with pMIG-*Rad52* or an empty pMIG retroviral vector as control, cultured them with LPS plus IL-4 for 96 h, before analysing CSR. Enforced Rad52 expression in *Rad52*^*+/+*^*Ku86*^*+/+*^ B cells increased Rad52 protein level by 2.4-fold without altering Ku86 protein expression and reduced by >65% CSR to IgG1 (Fig. 8a,c). Thus, Rad52 (in excess) competed with Ku70/Ku86 for binding to S-region DSB ends, thereby skewing the DSB synaptic process towards intra-S region recombination and significantly decreasing CSR.

### Switching B cells decrease *Rad52* and increase *Ku70/Ku86*

**Figure 9:**
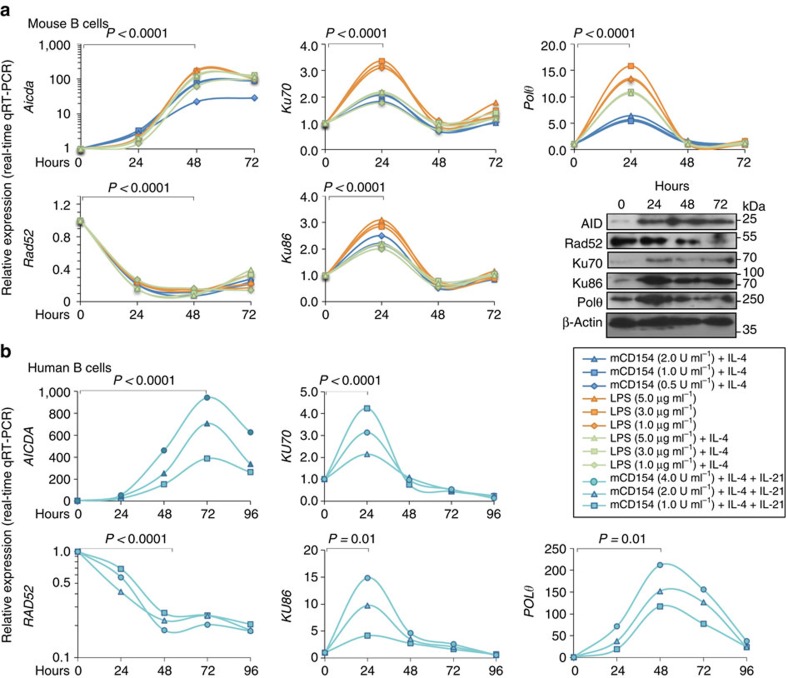
*Aicda* (*AICDA*)/CSR-inducing stimuli decrease expression of *Rad52* (*RAD52*) and increase expression of *Ku70/Ku86* (*KU70/KU86*) and *Polθ* (*POLθ*) in mouse and human B cells. (**a**) C57BL/6 B cells were stimulated with different amounts of LPS only, or LPS or mCD154 plus IL-4 for 0, 24, 48 and 72 h. *Aicda*, *Rad52*, *Polθ* and *Ku70*/*Ku86* transcripts were analysed by real-time qRT–PCR. Expression of *Aicda* normalized to *Cd79b* expression, expression of *Rad52*, *Ku70/Ku86* and *Polθ* normalized to *Gapdh* expression and depicted relative to the expression in unstimulated B cells, set as 1. Expression of AID, Rad52, Ku70, Ku86, Polθ and β-Actin proteins in B cells stimulated with LPS (3.0 μg ml^−1^) plus IL-4 were analysed by immunoblotting. (**b**) Human naive B cells were stimulated with different amounts of mCD154 plus human IL-4 and human IL-21 for 0, 24, 48, 72 or 96 h. *AICDA*, *RAD52*, *KU70*/*KU86* and *POLθ* transcripts were analysed by real-time qRT–PCR. Expression normalized to *CD79b* expression and depicted relative to the expression in unstimulated B cells, set as 1. *P*-values determined using a paired Student's *t*-test. Data are from one representative of three independent experiments.

Given the reciprocal modulation of CSR by Rad52 and Ku70/Ku86, we hypothesized that, on exposure to CSR-inducing stimuli, B cells downregulate Rad52 and upregulate Ku70/Ku86 to reduce intra-S region and facilitate inter-S–S region recombination, thereby ensuring maximal CSR rates. Indeed, in B cells stimulated with increasing amounts of LPS and LPS or mCD154 plus IL-4, which induced *Aicda* and CSR, *Rad52* transcript levels were reduced by 73%–96% (*P<*0.0001, paired Student's *t*-test) after 24–48 h of culture (Fig. 9a). In contrast to *Rad52*, *Ku70* and *Ku86* transcripts were increased by two- to threefolds (*P=*0.00013 or *P<*0.0001, respectively, paired Student's *t*-test) within 24 h and returned to baseline values by 48 h. *Polθ* followed the upregulation/downregulation kinetics of *Ku70/Ku86*. The expression of Rad52, Ku70/Ku86, AID and Polθ proteins followed tightly with the kinetic of expression of their corresponding transcripts. Rad52 protein was downregulated on stimulation by LPS plus IL-4, whereas Ku70/Ku86 and Polθ proteins were upregulated. Similar to mouse B cells, human B cells also displayed a reciprocal modulation of *RAD52* and *POLθ*, *KU70*, *KU86* by *AICDA*/CSR-inducing stimuli (mCD154 plus IL-4 and IL-21), with the exception of a delayed recovery of *RAD52* expression (Fig. 9b). Thus, B cells induced to undergo CSR modulate *Rad52* (*RAD52*) and *Ku70/Ku86* (*KU70*/*KU86*) expression in a reciprocal manner at both transcription and protein levels, thereby skewing the S-region DSB synaptic process towards inter-S–S region recombination.

## Discussion

Resolution of DSBs is a highly coordinated process that uses the DNA repair machinery to maintain genomic integrity. DSBs are generally repaired by HR or NHEJ—NHEJ is also referred to as classical-NHEJ. HR accurately repairs staggered post-replicative DSBs using a long, homologous single-strand template in the form of a sister chromatid, whereas NHEJ fuses blunt or virtually blunt DSB ends that lack substantial joining complementarity, to form ‘direct' junctions^10^,^12^. Rad52 and Ku70/Ku86 are critical and early elements in HR and NHEJ DSB, respectively. Both Rad52 and Ku70/Ku86 bind to DSB ends and facilitate end-to-end interactions^43^. Rad52 binds to and wraps around resected DSBs, thereby efficiently promoting annealing of complementary DNA single strands^31^, although it can also bind to blunt DSB ends^43^,^44^,^45^. Ku70/Ku86 binds to blunt DSB ends and gives rise to ‘clean' (that is, free of microhomologies) DSB junctions, although it can also bind, albeit less efficiently, to DSBs with relatively short protruding ends^44^,^46^.

Efficient inter-S–S region recombination that leads to CSR requires DSB resolution by NHEJ, which recombines S-region DSBs to form S–S junctions with no or minimal microhomologies^10^,^11^,^12^. Substantial CSR, however, occurs in the absence of core NHEJ components by A-NHEJ, which yields S–S junctions with microhomologies^12^,^16^,^17^,^18^. In addition to CSR, microhomology-mediated A-NHEJ would also effect oncogenic inter-chromosomal translocations, which are indeed characterized by DSB junctions with significant levels of microhomologies^8^,^25^. Here we show that the SSA and HR element Rad52 plays a central role in facilitating the A-NHEJ DSB synaptic process underpinning CSR, as well as t(15;12) *c-Myc/IgH* translocation. Rad52 functions as a recombination mediator and is involved in maintenance of genomic integrity. It interacts with replication protein A^47^, another single-strand DNA-binding protein, which plays a role in CSR. Replication protein A associates preferentially with resected single-strand DNA ends created by AID, thereby helping stabilize AID itself^48^.

Extensive resection of DSBs yielding long overhangs (200 nt to 2–4 kb)^8^,^9^ leads to HR, whereas less extensive DSB resection (generally >30 but <200 nt)^20^,^32^ promotes what is referred to as SSA pathway, which is mediated by Rad52 but does not require a sister chromatid template^49^,^50^. More limited (<30 nt) DSB resection facilitates single-strand DNA annealing leading to MMEJ^9^,^20^,^48^, suggesting that the distinction between SSA and MMEJ is based on the length of the complementary protruding ends involved in the synaptic process. The requirement for Rad52 in SSA but not MMEJ has been claimed to be another criterion to distinguish SSA from MMEJ^21^. Data in fission yeast, however, suggest an extensive overlap between SSA and MMEJ, as effected by Rad22, the fission yeast homologue of Rad52 (refs 23, 27). Other reports have attributed contradictory roles to Rad52 in MMEJ. Rad52 has been shown to suppress MMEJ in budding yeast, possibly by promoting Rad51 filament formation and thereby HR^51^,^52^, but it has also been shown to be required for MMEJ in both budding and fission yeast^22^,^23^,^53^. DSB junctional microhomologies in *yku70/80*Δ*rad52*Δ budding yeast cells were thought to indicate an independence of MMEJ from Rad52. Such microhomologies, however, were likely to be introduced by Rad59, a Rad52 homologue that anneals single-strand DNAs with short homologous sequences^23^,^27^,^45^,^54^.

By virtue of its low-fidelity translesion DNA synthesis, Polθ plays an important role, as we have shown, in SHM^37^. *Polθ*^*−/−*^ B cells were comparable to their *Polθ*^+/+^ counterparts in switching to IgG1, IgG3, IgG2c and IgA, as well as in frequency and extent of Sμ–Sγ1 junctional microhomologies. These findings confirm and extend a previous observation^40^, and further suggest that Polθ does not play a role in Ku-independent (microhomology-mediated) A-NHEJ of CSR. This is in spite of the ability of Polθ to promote annealing of resected DSBs and facilitate MMEJ^32^,^34^,^35^,^36^. The failure of Polθ deficiency to alter CSR would not necessarily rule out other contributions by Polθ to this process, such as insertion of mutations into inter-S–S junction surrounding areas, perhaps consistent with the upregulation of Polθ in induced B cells, as we have previously shown and confirmed here^1^,^37^. Such an upregulation of Polθ may be part of an overall ‘early' repair response, which includes primarily Ku70/Ku86, to the DNA damage associated with CSR and probably SHM. The apparent lack of an obvious role of Polθ in CSR might also reflect a functional redundancy of Polθ in this process, as other DNA polymerases, such as human Polλ and Polβ, have been shown to be interchangeable in mediating DNA single-strand annealing and elongation^55^—Polλ in conjunction with Polδ also plays a role in *Saccharomyces cerevisiae* MMEJ^52^. Redundancy in A-NHEJ would not be limited to Polθ, as deficiency of Lig1 or Lig3 (the DNA ligases involved in A-NHEJ) alone did not affect CSR, possibly due to the functional interchangeability of these two ligases in the late stage of CSR^56^.

Unlike Polθ, Rad52 plays an important role in CSR. Similar to Ku70/Ku86, Rad52 was recruited to CSR-targeted S regions after AID processing. Increased CSR in the absence of Rad52 entailed enhanced recruitment of Ku70/Ku86 to S-region DSB ends, decreased intra-Sμ DSB recombinations, reduced frequency and length of microhomologies in S–S junctions, as well as decreased inter-chromosomal *c-Myc*/*IgH* translocations. All of these features are opposite to those in (NHEJ-deficient) *Ku70*^*−/−*^ B cells, which showed increased intra-S region DSB recombination, increased frequency and length of microhomologies in S–S junctions, as well as increased inter-chromosomal *c-Myc*/*IgH* translocations^16^,^17^,^18^, and point at Rad52 as mediator of A-NHEJ in CSR (Supplementary Table 1)^17^,^18^. Thus, our findings argue for a role of Rad52 in a Ku-independent DSB synaptic process that gives rise to S–S junctions. These contain microhomologies, which are more abundant than those, if any, of Ku-dependent S–S synapses^16^,^17^,^18^.

Recruitment of Rad52 or Ku70/Ku86 to DSB ends would lead to different synaptic processes^43^. Rad52 preferentially synapsed DSB ends within the same S region, as emphasized by the greatly decreased intra-Sμ region deletions, which are marks of intra-S region DNA recombination or ‘rejoining', in *Rad52*^*−/−*^ B cells. Conversely, intra-S region deletions were found to be greatly increased in B cells lacking Ku70 or Ku70 and Lig4 (ref. 17), as well as in B cells lacking 53BP1, a DNA-damage response protein that favours long-range S–S recombination (leading to CSR), but prevents short-range rejoining of intra-S region DSBs, possibly because of its protection of DNA ends from resection^57^. Rad52 recruitment to CSR-targeted Sγ was as efficient as recruitment to Sμ region. However, because of the distinctive, albeit characteristically repetitive, sequences of the *IgH* locus S regions, DSB protruding ends in two different S regions, such as Sμ and Sγ1, are less suitable substrates for Rad52-mediated annealing than DSB protruding ends in the same S region. Thus, although (Ku-dependent) NHEJ skewed the DSB repair process towards inter-S–S region recombination, thereby increasing CSR rates, Rad52-dependent annealing of DNA single strands favoured DSB synapsing within the same S region, which led to intra-S region recombinations, thereby lowering CSR rates. The latter process was further emphasized by the dampening of CSR on enforced expression of Rad52.

We show here that, similar to Ku70/Ku86, Rad52 bound to the DSB free ends of CSR-targeted S regions, but did not bind to S regions not involved in CSR or Cμ region. By binding to S-region DSB free ends, Rad52 prevented Ku70/Ku86 from accessing such DSB ends, as shown by the specific ChIP assays involving anti-Ku70/Ku86 monoclonal antibody and *Rad52*^*−/−*^ B cells, the specific competition assays using recombinant Rad52 and Ku70/Ku86 proteins, as well as the supershift EMSAs involving anti-Ku70/Ku86 monoclonal antibody and nuclear extracts from *Rad52*^*−/−*^ B cells. The competition of Rad52 with Ku70/Ku86 for binding to S-region DSB ends was further emphasized by the decreased CSR on enforced expression of Rad52 in (normal) B cells. Such CSR decrease was more pronounced than that resulting from knocking down Ku86 in normal B cells. In these B cells, Ku86 was present in residual amounts but competed with lower amounts of Rad52 than in B cells in which Rad52 was enforced expressed. The virtual ablation of (the otherwise much increased) CSR by Ku86 knockdown in *Rad52*^*−/−*^ B cells not only further emphasized the critical role of the Ku70/Ku86 heterodimer in the inter-S–S region DSB synaptic process but also revealed a contribution of Rad52 to inter-S–S region recombination and CSR.

In B cells induced to undergo CSR, execution of the DSB synaptic process towards inter-S–S region recombination would be further facilitated by the downregulation of *Rad52* (*RAD52*) with concomitant upregulation of *Ku70/Ku86* (*KU70/KU86*) together with *Aicda* (*AICDA*) and *Polθ* (*POLθ*) transcripts at early time points (24–48 h). The expression of Rad52, Ku70/Ku86, AID and Polθ proteins followed tightly the kinetic expression of their corresponding transcripts. This was particularly the case for Rad52, which is a short-lived protein^58^, in contrast to the relatively stable Ku70/Ku86. In switching B cells, *Rad52* (*RAD52*)/Rad52 downregulation was profound but not complete, possibly allowing for some residual intra-S recombination. After 48–72 h, the return of *Ku70/Ku86* (*KU70/KU86*) transcripts and Ku70/Ku86 proteins to a lower expression levels would be important to maintain a Rad52:Ku70/Ku86 ratio that prevents CSR dysregulation. Thus, the modulatory changes in Rad52 and Ku70/Ku86 are likely to be the result of CSR induction rather than a consequence of cell proliferation.

As we showed here, Rad52 deficiency did not alter B cell cycle or B-cell division and increased CSR independently of cell division. Because of its use of sister chromatids as templates to mediate faithful repair, HR is generally restricted to S and G2 phases. NHEJ and MMEJ generally operate in G0–G1^8^, although both can function throughout the whole cell cycle^8^. In yeast, Rad52 is generally recruited to DSBs and readily form foci in response to DNA damage in S and G2/M, when a sister chromatid template becomes available to guide the repair process^59^. Rad52, however, can also accumulate at DNA damage sites immediately after insertion of DSBs outside the S and G2/M phases^60^,^61^, thereby promoting annealing of single-strand DNA and leading to (micro)homology-mediated end-joining in the absence of a sister chromatid template. Furthermore, HR has been suggested to contribute to S-region DSB repair and CSR, in addition to NHEJ/A-NHEJ^48^,^62^. Indeed, DSBs not processed by NHEJ or A-NHEJ in G0–G1 would be further resected in S-G2/M and repaired by HR using the second (intact) *IgH* allele as a template^48^. Thus, given the role of Rad52 in both A-NHEJ and HR, the contribution of Rad52 to S region DSB repair would extend throughout the whole cell cycle. Rad52-facilitated microhomology-mediated A-NHEJ may complement NHEJ in the presence of high DSB rates, such as those in hypermutating/switching GC B cells, thereby functioning as a salvage pathway for DSBs that are not repaired by NHEJ.

Collectively, our findings show that Rad52 competes with Ku for binding to S-region DSB free ends, where it facilitates a DSB synaptic process, which favours intra-S region recombination, and also mediates, in particular in the absence of a functional NHEJ pathway, inter-S–S region recombinations (Supplementary Fig. 5).

## Methods

### Mice

*Rad52*^*−/−*^ mice were generated by Dr Albert Pastink (Leiden University, Leiden, The Netherlands) by replacing exon 3 of the *Rad52* gene with positive selection marker neomycin, as driven by the phosphoglycerate kinase promoter, and an upstream mouse sequence functioning as a transcription terminator^39^. These mice were backcrossed to C57/BL6 mice for more than six generations. No full-length or truncated Rad52 protein was produced from the disrupted allele. *Rad52*^*−/−*^ mice were viable and fertile, and showed no gross abnormalities^39^. *p53*^*−/−*^ (B6.129S2-*Trp53*^*tm1Tyj*^/J) mice were purchased from Jackson Laboratory (Bar Harbor, Maine). *p53*^*−/−*^*Rad52*^*+/+*^ and *p53*^*−/−*^*Rad52*^*–/*–^ mice were generated in our facilities by cross-breeding *p53*^*−/−*^ with *Rad52*^*+/−*^ mice. *Aicda*^*−/−*^ mice (C57BL/6 background)^63^ were obtained from Dr Tasuku Honjo (Kyoto University, Kyoto, Japan). *Polθ*^−/−^ mice (C57BL/6 background) were generated by Dr John Schimenti (Cornell University, Ithaca, NY) by placing an in-frame stop codon into exon 1 and replacing exons 2–5 with a neomycin resistance (*neo*) gene^37^. All mice were housed in pathogen-free conditions. Both male and female mice aged 8–12 weeks were used for the experiments. The Institutional Animal Care and Use Committee of the University of Texas Health Science Center at San Antonio approved all animal protocols.

### NP_16_-CGG and OVA immunization and antibody titration

*Rad52*^+/+^, *Rad52*^−/−^, *Polθ*^+/+^ and *Polθ*^−/−^ mice (8–12 weeks of age) were injected intraperitoneally (i.p.) with 100 μg of NP_16_-CGG (average 16 molecules of 4-hydroxy-3-nitrophenyl acetyl coupled to 1 molecule of chicken γ-globulin; Biosearch Technologies) in 100 μl of alum (Imject Alum, Thermo Fisher Scientific). Serum was collected 10 days later for titration of circulating total and NP-binding IgM and IgG1 using enzyme-linked immunosorbent assays, as we described^64^,^65^,^66^. To analyse the impact of Rad52 deficiency in IgE response, *Rad52*^+/+^ and *Rad52*^−/−^ mice were injected i.p. with 20 μg of OVA in 100 μl of alum and ‘boost' injected 7 days later with 20 μg of OVA in PBS. Serum was collected before or 5 days after the ‘boost' injection for titration of circulating total IgE.

### B and T cells, cell cycle and proliferation

B cells (B220^+^), CD4^+^ and CD8^+^ T cells, dead B cells, GC (B220^+^PNA^hi^) B cells and plasma cells (B220^lo^CD138^+^) were identified and analysed by flow cytometry^64^,^65^,^67^ using a FACSCalibur or LSR-II flow cytometer (BD Biosciences). Data analysis was performed using FlowJo software (Tree Star). Single-cell suspensions were prepared from spleens of *Rad52*^+/+^ and *Rad52*^−/−^ mice, and stained with PE-anti-B220 monoclonal antibody (clone RA3-6B2, eBioscience, 0.05 μg ml^−1^), fluorescein isothiocyanate (FITC)-anti-CD3 monoclonal antibody (clone 17A2, BioLegend, 0.1 μg ml^−1^), FITC-anti-CD4 (clone GK1.5, BioLegend, 0.1 μg ml^−1^) monoclonal antibody and/or allophycocyanin (APC)-anti-CD8 monoclonal antibody (53-6.7, BD Biosciences, 0.1 μg ml^−1^), Alexa Fluor 647-peanut agglutinin (PNA; Invitrogen) and biotin-anti-CD138 monoclonal antibody (clone 281-2, BD Biosciences, 0.05 μg ml^−1^) followed by FITC-streptavidin (11-4317-87, eBioscience, 0.5 μg ml^−1^) or PE-streptavidin (12-4317-87, eBioscience, 0.1 μg ml^−1^), PE-anti-IgM monoclonal antibody (clone AF6-78, BD Biosciences, 0.1 μg ml^−1^), FITC-anti-IgG1 monoclonal antibody (clone A85-1, BD Biosciences, 0.1 μg ml^−1^), FITC-anti-IgG3 monoclonal antibody (R40-82, BD Biosciences, 0.1 μg ml^−1^), FITC-anti-IgA monoclonal antibody (clone C10-3, BD Biosciences, 0.1 μg ml^−1^) and biotin-rat-anti-mouse IgG2a/c monoclonal antibody (clone R19-15, BD Biosciences, 0.1 μg ml^−1^), followed by APC-streptavidin (550874, eBioscience, 0.5 μg ml^−1^) and 7-AAD (BD Biosciences, 0.25 μg per 1 × 10^6^ cells). For analysis of B-cell proliferation and cell cycle *in vivo*, mice were immunized with NP_16_-CGG. Ten days after NP_16_-CGG immunization, the mice were injected i.p. with BrdU (1 mg in 200 μl PBS) twice within a 16 h interval and killed 4 h after the last injection. Spleen cells stained with PE-anti-B220 monoclonal antibody and APC-anti-BrdU monoclonal antibody (APC BrdU Flow Kit, BD Biosciences) to detect intracellular BrdU were analysed by flow cytometry. To analyse cell cycle, B220^+^ B cells or B220^+^PNA^hi^ GC B cells were stained for surface B220 and PNA with PE-anti-B220 monoclonal antibody (clone RA3-6B2, eBioscience, 0.05 μg ml^−1^) and Alexa Fluor 647-labelled PNA (Invitrogen), fixed/permeabilized and stained with APC-anti-BrdU monoclonal antibody and 7-AAD (APC BrdU Flow Kit; BD Biosciences), and then analysed by flow cytometry. B-cell division was analysed by carboxyfluorescein succinimidyl ester (CFSE) dilution using the CellTrace CFSE Cell Proliferation Kit (Invitrogen). B cells were incubated for 5 min at 37 °C in 3 ml PBS with 2.5 μM CFSE at a density of 1 × 10^7^ cells per ml and then washed in fetal bovine serum (FBS)–RPMI. Cells were then cultured in the presence of LPS plus IL-4 or membrane fragments of baculovirus-infected Sf21 insect cells expressing mouse CD154 (ref. 68) (mCD154, 1 or 2 U ml^−1^) plus IL-4 for 72 h, before being stained with PE-anti-B220 monoclonal antibody, 7-AAD and APC-anti-IgG1 monoclonal antibody (clone X56, BD Biosciences, 0.1 μg ml^−1^), and analysed by flow cytometry.

### CSR analysis *in vitro* and *in vivo*

B cells isolated from red blood cell-depleted mouse splenocytes were purified by negative selection of cells expressing CD43, CD4, CD8, CD11b, CD49b, CD90.2, Gr-1 or Ter-119 using the EasySep Mouse B-cell Isolation kit (StemCell Technologies). B cells were resuspended in RPMI 1640 medium with 10% FBS, 50 mM β-mercaptoethanol and 1 × antibiotic–antimycotic mixture (15240-062; Invitrogen) (FBS–RPMI) at 37 °C in 48-well plates and stimulated with the following reagents: LPS (3 μg ml^−1^) from *Escherichia coli* (055:B5; Sigma-Aldrich) for CSR to IgG3; LPS (3 μg ml^−1^) or mCD154 (1 U ml^−1^) plus IL-4 (5 ng ml^−1^; R&D Systems) for CSR to IgG1; LPS (3 μg ml^−1^) plus TGF-β (2 ng ml^−1^; R&D Systems), IL-4 (5 ng ml^−1^) and IL-5 (3 ng ml^−1^) (R&D Systems), and anti-IgD monoclonal antibody/dextran (anti-δ monoclonal antibody/dex) (Fina Biosolutions) for CSR to IgA; LPS (3 μg ml^−1^) plus IFN-γ (50 ng ml^−1^) (R&D Systems) for CSR to IgG2a/c. After 96 h, B cells were stained with FITC-labelled rat monoclonal antibody to mouse IgG1 (clone A85-1, 0.1 μg ml^−1^), mouse IgG2a/c (clone R19-15, 0.1 μg ml^−1^), mouse IgG3 (clone R40-82, 0.1 μg ml^−1^), mouse IgA (clone C10-3, 0.1 μg ml^−1^) or PE-labelled rat monoclonal antibody to mouse B220 (clone RA3-6B2, 0.05 μg ml^−1^; all from BD Biosciences). Stained cells were analysed by flow cytometry. To analyse *in vivo* CSR, spleen or Peyer's patch B cells were isolated from *Rad52*^+/+^ and *Rad52*^−/−^ mice 10 days after immunization with NP_16_-CGG, and were stained with PE-labelled anti-B220 monoclonal antibody or Alexa Fluor 647-labelled PNA, as well as FITC-labelled anti-IgG1 (clone A85-1, 0.1 μg ml^−1^) or FITC-labelled anti-IgA (clone C10-3, 0.1 μg ml^−1^) monoclonal antibodies (BD Biosciences), and then analysed by flow cytometry.

### Human B cells and stimulation

Human naive B cells (>95% pure) were purified by negative selection, using the EasySep Human Naive B Cell Enrichment Kit (19254; StemCell Technologies), from healthy donor peripheral blood mononuclear cells, following the manufacturer's instructions. Naive B cells were then cultured in FBS–RPMI and stimulated with mCD154 (1, 2 or 4 U ml^−1^), recombinant human IL-4 (20 ng ml^−1^; R&D Systems) and recombinant human IL-21 (50 ng ml^−1^; R&D Systems) for 24, 48, 72 and 96 h. RNA was extracted from stimulated and unstimulated B cells and used for qRT–PCR analysis.

### qRT–PCR analysis of transcripts

Total RNA was extracted from 2–5 × 10^6^ B cells using the RNeasy Mini Kit (Qiagen). Residual DNA was removed using genomic DNA eliminator columns (Qiagen). cDNA was synthesized from 1 to 2 μg total RNA with the SuperScript III First-Strand Synthesis System (Invitrogen) using oligo-dT primer. The expression of mouse germline Iγ1-Cγ1 and Iγ3-Cγ3, circle Iγ1-Cμ and Iγ3-Cμ, post-recombination Iμ-Cγ1 and Iμ-Cγ3, *Aicda*, *Rad52*, *Ku70*, *Ku86*, *Polθ*, *Cd79b* and *Gapdh* transcripts, as well as human *AICDA*, *RAD52*, *POLθ*, *KU70*, *KU86* and *CD79b* transcripts, was measured by real-time qRT–PCR with appropriate primers^3^,^64^,^65^ (Supplementary Table 2). A Bio-Rad MyiQ Real-Time PCR Detection System (Bio-Rad Laboratories) was used to measure SYBR Green (IQ SYBR Green Supermix, Bio-Rad Laboratories) incorporation with the following protocol: 95 °C for 15 s, 40 cycles of 94 °C for 10 s, 60 °C for 30 s, 72 °C for 30 s. Data acquisition was performed during this 72 °C extension step. Melting curve analysis was performed from 72 to 95 °C. The change in cycling threshold (ΔΔCt) method was used to analyse levels of transcripts and data were normalized to the level of *Cd79b/CD79b*, which encodes the BCR *Igβ* chain constitutively expressed in B cells, or *Gapdh*.

### Immunoblotting

B cells were lysed in Laemmli buffer. Cell extracts containing equal amounts of protein (20 μg) were fractionated through SDS–PAGE (10%). The fractionated proteins were transferred onto polyvinylidene difluoride membranes (Bio-Rad) overnight (30 V/90 mA) at 4 °C. After blocking and overnight incubation at 4 °C with anti-AID antibody (H-80, Santa Cruz, 0.2 μg ml^−1^), anti-Ku70 antibody (C-19, Santa Cruz, 0.2 μg ml^−1^), anti-Ku86 monoclonal antibody (clone 111, Thermo Fisher Scientific, 0.2 μg ml^−1^), anti-Rad52 antibody (H-300, Santa Cruz Biotechnology, 0.4 μg ml^−1^), anti-Polθ antibody (AAS04492C, Antibody Verify, 1 μg ml^−1^) or anti-β-Actin monoclonal antibody (2F1-1, BioLegend, 0.1 μg ml^−1^), the membranes were incubated with horseradish peroxidase (HRP)-conjugated secondary antibodies. After washing with TBS–Tween 20 (0.05%), bound HRP-conjugated antibodies were detected using Western Lightning Plus-ECL reagents (PerkinElmer Life and Analytical Sciences).

### Semi-quantitative digestion–circularization PCR

B-cell genomic DNA was digested with HindIII before being ligated with T4 DNA ligase to generate circularized DNA^65^. Sμ–Sγ1 and Sμ–Sγ3 HindIII fragments were amplified by nested PCR using Phusion High-Fidelity DNA polymerase (New England Biolabs) with reported primers^69^. The completion of HindIII digestions and ligations was verified by PCR with a primer set specific for *Gapdh*. The first round PCR was carried out for 25 cycles at 94 °C for 30 s, 58 °C for 30 s, 72 °C for 35 s. Product (1 μl) of the first-round PCR was used as template for the second round of PCR for 30 cycles at 94 °C for 30 s, 60 °C for 30 s, 72 °C for 30 s.

### DNA S–S junctional sequences

Genomic DNA was prepared from B cells cultured for 96 h with LPS and rmIL-4, or B220^+^PNA^hi^ B cells from Peyer's patches. Sμ–Sγ1 or Sμ–Sα DNA was amplified by two sequential rounds of specific PCR using Phusion high-fidelity DNA polymerase (New England Biolabs) and nested oligonucleotide primers^41^,^67^ (Supplementary Table 2). The first and second rounds of PCR were performed at 94 °C for 45 s, 55 °C for 45 s, 72 °C for 4 min (30 cycles). PCR products were purified using a QIAquick PCR purification kit (Qiagen) and cloned into the pCR-Blunt II-TOPO vector (Invitrogen) for sequencing. Sequence alignment was performed by comparing the sequences of PCR products with Sμ and Sγ1 or Sα genomic sequences using National Center for Biotechnology Information BLAST (www.ncbi.nih.gov/BLAST).

### ChIP assays

ChIP assays were performed as described^64^,^65^,^70^. B cells (1 × 10^7^) were treated with formaldehyde (1% v/v) for 10 min at 25 °C to cross-link chromatin. After quenching with 100 mM of glycine (pH 8.0) and washing with cold PBS containing protease inhibitors (Roche), B cells were suspended in lysis buffer (20 mM Tris-HCl, 200 mM NaCl, 2 mM EDTA, 0.1% w/v SDS and protease inhibitors pH 8.0). Chromatin was sonicated to yield DNA fragments (∼200 to 1,000 bp in length), pre-cleared with Pierce Protein A beads (Thermo Fisher Scientific) and then incubated with rabbit anti-Rad52 antibody (H-300; Santa Cruz Biotechnology, 5 μg ml^−1^), anti-Ku70/86 monoclonal antibody (MA1-21818, Thermo Fisher Scientific, 5 μg ml^−1^), or control rabbit or mouse IgG with irrelevant specificities at 4 °C overnight. Immune complexes were precipitated by Protein A agarose beads, washed and eluted (50 mM Tris-HCl, 0.5% SDS, 200 mM NaCl, 100 μg ml^−1^ proteinase K pH 8.0), followed by incubation at 65 °C for 4 h. DNA was purified using a QIAquick PCR purification kit (Qiagen) and used as a template for analysis of Sμ, Sγ1 and Sγ3 by quantitative PCR using specific primers^70^ (Supplementary Table 2).

### *In situ* DNA end-labelling with biotin

DNA DSB ends were labelled with biotin^41^. *Rad52^+/+^* and *Rad52^−/−^* B cells were stimulated with LPS or LPS plus IL-4 for 60 h. Live B cells were separated through a Ficoll gradient, followed by fixation, permeabilization and *in situ* DNA end-labelling with bio-dUTP using TdT (Fig. 6d). Chromatin was pulled down with rabbit anti-Rad52 antibody (H-300, Santa Cruz Biotechnology, 5 μg ml^−1^), anti-Ku70/Ku86 monoclonal antibody (MA1-21818, Thermo Fisher Scientific, 5 μg ml^−1^) or control rabbit or mouse IgG with irrelevant specificity. Genomic DNA was prepared using a QIAquick PCR purification kit (Qiagen). Biotin-labelled DNA fragments were captured by streptavidin magnetic beads (Promega) before being analysed by quantitative PCR.

### Electrophoretic mobility shift assays

An ‘asymmetrical' biotin-labelled double-strand Sμ DNA probe was generated by PCR using a synthetic oligonucleotide Sμ1F, which consisted of part of the mouse Sμ region sequence 5′-CATTAATCTAGGTTGAATAGAGCTAAACTCTACTGCCTACACTGGACTGTTCTGAGCTGAGATGAGCTGGGGTG-3′ as template, and amplified with forward Sμ3F-Kpn (5′-AATTCGGTACCAACTTCATTAATCTAGGTTGAATAG-3′) primer, which included a KpnI site, and a Sμ3R (5′-CACCCCAGCTCATCTCAGC-3′) primer. Bio-dUTP was added to the PCR reaction. The PCR product was treated with T4 DNA polymerase in the presence of dNTP and digested by KpnI to generate a 4-nt 3′-single-strand overhang at one end of the oligonucleotide, the other end being blunt. The biotin-labelled probe was purified with the QIAquick Gel Extraction Kit (Qiagen) and used for EMSA reactions (5 fmol per reaction). EMSA assays were performed using the LightShift Chemiluminescent EMSA Kit (Thermo Fisher Scientific) according to the manufacturer's instructions. For the Rad52–Ku70/Ku86 competition experiments, purified recombinant human RAD52 (Axxora) or KU70/KU86 (Cell Sciences) proteins were used alone (0.32 pmol), or mixed at a 1:1 ratio (0.32 pmol RAD52 and 0.32 pmol KU70/KU86), 2:1 (0.64 pmol RAD52 and 0.32 pmol KU70/KU86) or 4:1 (1.28 pmol RAD52 and 0.32 pmol KU70/KU86). In supershift experiments, nuclear extracts (10 μg protein) from *Rad52*^+/+^ and *Rad52*^−/−^ B cells stimulated with LPS plus IL-4 for 60 h were pre-incubated for 30 min at room temperature with anti-Ku70/Ku86 monoclonal antibody (MA1-21818, Thermo Fisher Scientific, 1 μg per reaction) or irrelevant mouse IgG before being incubated with the biotin-labelled Sμ probe. Electrophoresis of the DNA–protein complexes was carried out on a 5% non-denaturing polyacrylamide gel with 0.5 × TBE. The samples were then transferred to Hybond-N^+^ membranes with a semi-dry transfer followed by ultraviolet cross-linking. Detection was performed using the Chemiluminescent Nucleic Acid Detection Module (Thermo Fisher Scientific) according to the manufacturer's instructions.

### *c-Myc/IgH* translocations

Genomic DNA was isolated from *p53*^*−/−*^*Rad52*^*–/*–^ and *p53*^*−/−*^*Rad52*^*+/*+^ B cells stimulated with LPS plus IL-4 for 96 h. Nested PCRs for translocations were performed on genomic DNA from 1 × 10^6^ cells with GoTaq Long PCR Master Mix (Promega) using *IgH* forward and *c-Myc* reverse primers (Supplementary Table 2). PCR conditions were as follows: 95 °C for 2 min followed by 25 cycles (95 °C, 10 s; 62 °C, 45 s; and 65 °C, 6 min) for both the first and second rounds. Amplified DNA were fractionated through 1.0% agarose, blotted onto Hybond-N^+^ membranes and hybridized to biotin labelled *c-Myc*-specific oligonucleotide probe 5′-GACGCCACTGCACCAGAGACCCTGCAGCGATTCAG-3′ or *IgH*-specific oligonucleotide probe 5′-CCTGGTATACAGGACGAAACTGCAGCAG-3′ and detected using Streptavidin-HRP. To analyse the *c-Myc*–*IgH* junctional sequences, PCR products were treated with T4 DNA polymerase in the presence of dNTP, cloned into the pCR-Blunt II-TOPO vector (Invitrogen) and sequenced. Sequence alignment was performed by comparing the sequences of PCR products with germline *c-Myc* and *IgH* genomic sequences using National Center for Biotechnology Information BLAST.

### Analysis of intra-Sμ region recombination

To analyse intra-Sμ region DNA recombination, Sμ region DNA was amplified by nested PCR using mouse Sμ-specific primers (Supplementary Table 2) and GoTaq Long PCR Master Mix (Promega) from genomic DNA isolated from *Rad52*^+/+^ and *Rad52*^−/−^ B cells stimulated with LPS plus IL-4 for 96 h. Amplified DNA was treated with T4 DNA polymerase in the presence of dNTP and cloned into the pCR-Blunt II-TOPO vector (Invitrogen). Sμ region DNA of individual clones were re-amplified using the Sμ-specific primers (as above) and GoTaq Long PCR Master Mix. Amplified DNA was fractionated through 0.8% agarose gel. The intra-Sμ recombined DNAs, that is, Sμ region DNA with internal deletions, were detected by comparing the length of the amplified DNA with that of the respective germline Sμ region and positively identified by sequencing.

### Rad52 retroviral construct and enforced expression

*Rad52* cDNA was amplified from mouse B cells using the appropriate primers (Supplementary Table 2) and cloned into the pMIG retroviral expression vector (green fluorescent protein (GFP) translation initiated by the internal ribosome entry sites (IRES) in transduced B cells). To generate the retrovirus, pMIG vector encoding GFP or pMIG-Rad52 construct encoding GFP and Rad52, together with the pCL-Eco retrovirus-packaging vector (Imgenex), were used to transfect HEK293T cells by a Ca^++^ phosphate-mediated transfection procedure (ProFection Mammalian Transfection System, Promega). Viral supernatants were collected and used to transduce spleen B cells from C57BL/6 mice, as we reported^64^,^65^, after a 12 h LPS activation. Transduced B cells were then stimulated with LPS plus IL-4 for 96 h before analysis of GFP^+^ and IgG1^+^ B cells by flow cytometry^64^,^65^—dead (7-AAD^+^) cells were excluded from analysis. Expression of Rad52, Ku86 and β-Actin proteins in B cells transduced with empty or Rad52-expression pMIG retroviral construct were analysed by immunoblotting^66^.

### Ku86 knockdown using lentiviral shRNA construct

The Ku86-specific shRNA lentiviral construct pGFP-C-Ku86-shRNALenti (TL502435) and non-effective 29-mer scrambled shRNA lentiviral construct pGFP-C-scr-shLenti (TR30021) were obtained from Origene Technologies. To generate the lentivirus, pGFP-C-shLenti vector and packaging vectors were used to cotransfect HEK293T cells according to the manufacturer's instructions (Lenti-vpak Lentiviral Packaging Kit, Origene). Viral supernatants were collected and used to transduce *Rad52*^+/+^ and *Rad52*^−/−^ B cells. Transduced B cells were then stimulated with LPS plus IL-4 for 96 h before analysing GFP^+^ and IgG1^+^ B cells by flow cytometry—dead (7-AAD^+^) cells were excluded from analysis. Expressions of Rad52, Ku86 and β-Actin proteins in the transduced B cells were analysed by immunoblotting.

### Statistical analysis

Statistical analysis was performed using Excel (Microsoft), to determine *P*-values by paired and unpaired Student's *t-*tests; and *P*-values <0.05 were considered significant. Differences in the number and length of microhomologies in Sμ–Sγ1 and Sμ–Sα junctions between stimulated *Rad52*^+/+^ and *Rad52*^−/−^, or *Polθ*^+/+^ and *Polθ*^−/−^ B cells were analysed using the paired Student's *t*-test.

### Data availability

The data that support the findings of this study are available within the article and its Supplementary Information file or from the corresponding authors upon reasonable request.

## Additional information

**How to cite this article:** Zan, H. *et al*. Rad52 competes with Ku70/Ku86 for binding to S-region DSB ends to modulate antibody class-switch DNA recombination. *Nat. Commun.* **8**, 14244 doi: 10.1038/ncomms14244 (2017).

**Publisher's note:** Springer Nature remains neutral with regard to jurisdictional claims in published maps and institutional affiliations.

## Supplementary Material

Supplementary Information Supplementary Figures and Supplementary Tables
