# Supplementary material for: Rad52 competes with Ku70/Ku86 for binding to S-region DSB ends to modulate antibody class-switch DNA recombination
Source: Nat Commun. 2017 Feb 8;8:14244. doi: 10.1038/ncomms14244 (PMC5309807; doi:10.1038/ncomms14244)
Supplement: Supplementary Information — Supplementary Figures and Supplementary Tables [file ncomms14244-s1.pdf]

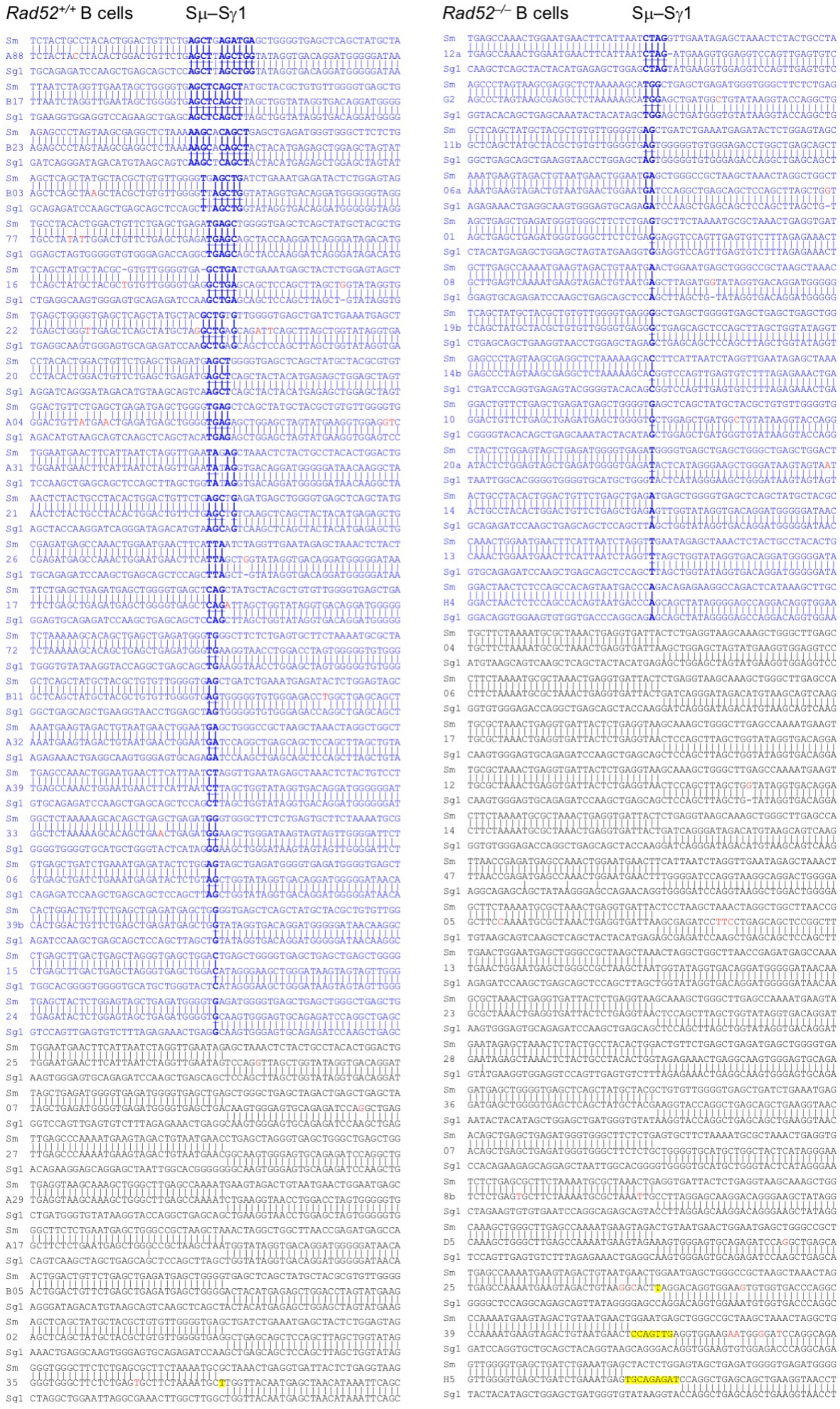

Supplementary Figure 1 | Rad52 deficiency reduces Sμ–Sγ1 junction microhomologies in CSR *in vitro*. B cells from *Rad52*$^{+/+}$ and *Rad52*$^{-/-}$ mice were stimulated with LPS plus IL-4 for 96 h. Sμ–Sγ1 Junctional DNAs from stimulated cells were amplified, cloned and sequenced. Thirty sequences from *Rad52*$^{+/+}$ B cells and 30 sequences from *Rad52*$^{-/-}$ B cells are shown. Each sequence is aligned with the corresponding germline Sμ (above) and Sγ1 (below) sequence. Microhomologies (bold and underlined) were determined by identifying the longest region at the Sμ–Sγ1 junction of perfect uninterrupted donor/acceptor identity or the longest overlap region at the S–S junction with no more than one mismatch on either side of the breakpoint. Sequences containing microhomologies are in blue; point-mutations in red; nucleotide insertions highlighted in yellow.

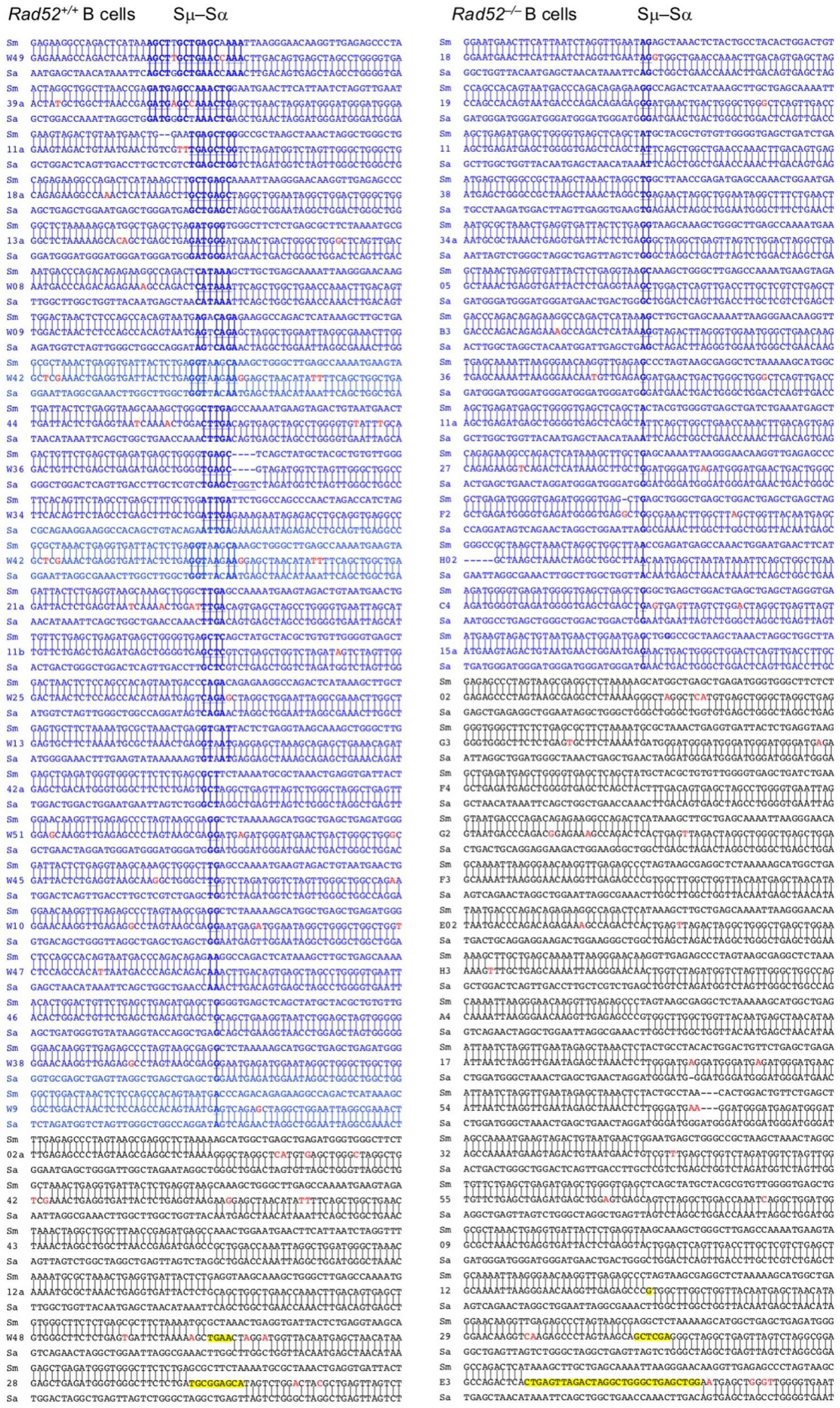

Supplementary Figure 2 | Rad52 deficiency reduces Sμ–Sα junction microhomologies in CSR *in vivo*. Sμ–Sα junctional DNAs from the Peyer's patch B cells of 3 *Rad52*$^{+/+}$ and 3 *Rad52*$^{–/–}$ mice were amplified, cloned and sequenced. Thirty sequences from *Rad52*$^{+/+}$ mice and 30 sequences from *Rad52*$^{–/–}$ mice are shown. Each sequence is aligned with the corresponding germline Sm (above) and Sa (below) sequence. Microhomologies (bold and underlined) were determined by identifying the longest region at the Sμ–Sα junction of perfect uninterrupted donor/acceptor identity or the longest overlap region at the switch junction with no more than one mismatch on either side of the breakpoint. Sequences containing microhomologies are in blue; point-mutations in red; nucleotide insertions highlighted in yellow.

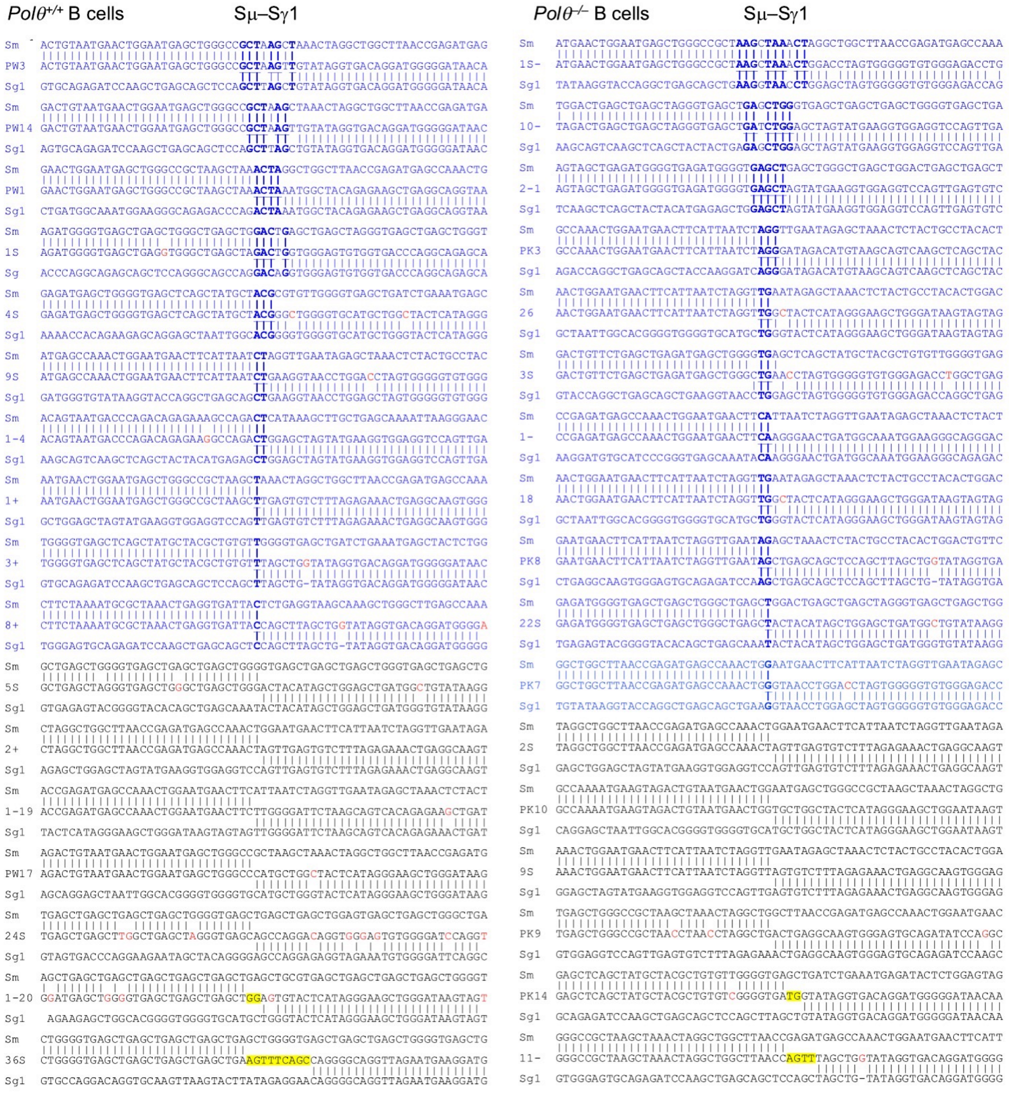

Supplementary Figure 3 | Polθ deficiency does not alter CSR Sμ–Sγ1 DNA junctions in B cells. *Polθ*$^{+/+}$ and *Polθ*$^{-/-}$ B cells were stimulated with LPS plus IL-4 for 96 h. Sμ–Sγ1 junctional DNAs from stimulated B cells were amplified, cloned and sequenced. Each sequence is aligned with the corresponding germline Sμ (above) and Sγ1 (below) sequence. Microhomologies are bold and underlined. Sequences were derived from stimulated B cells of two pairs of *Polθ*$^{+/+}$ and *Polθ*$^{-/-}$ mice. Sequences containing microhomologies are in blue; point-mutations in red; nucleotide insertions highlighted in yellow.

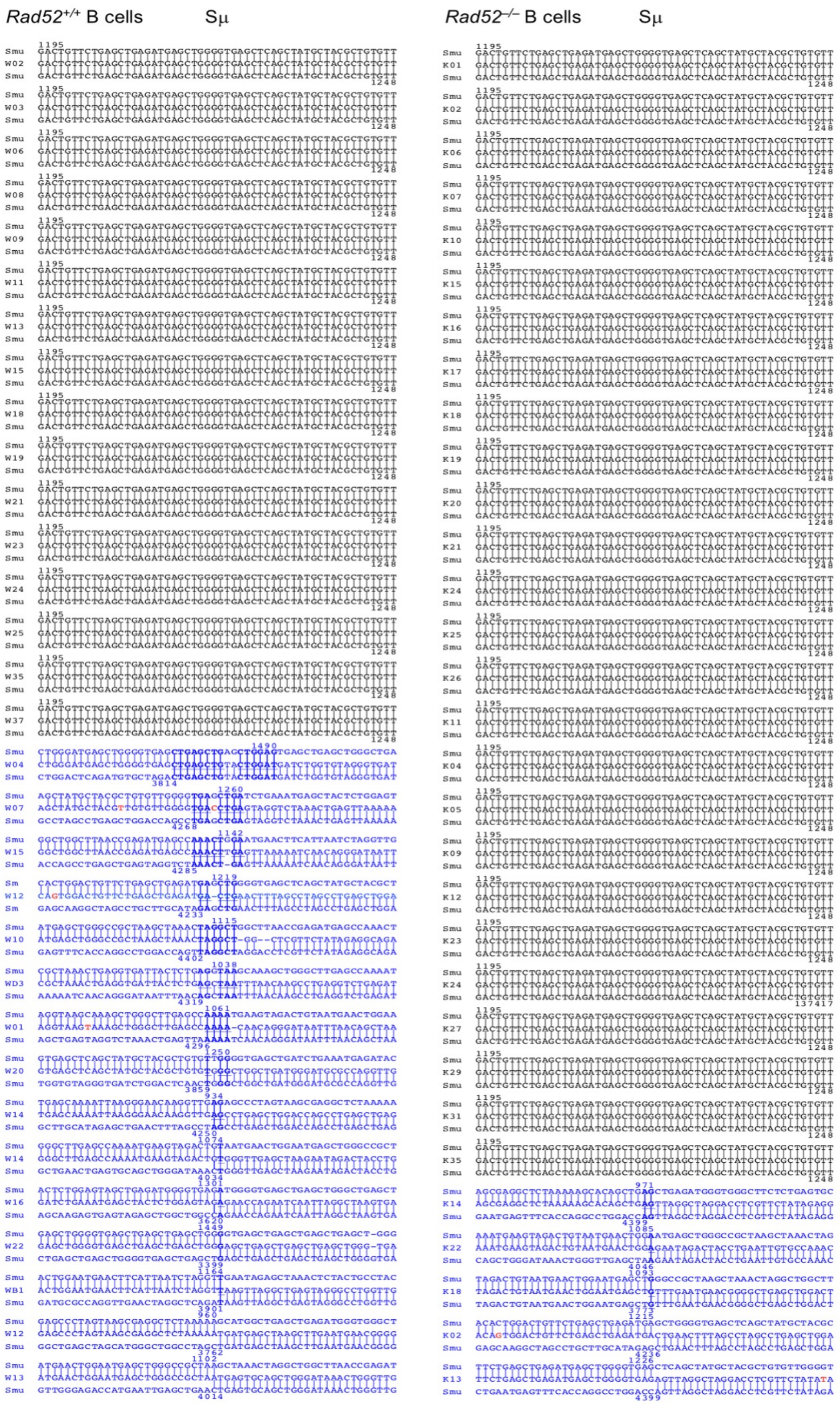

Supplementary Figure 4 | Rad52 deficiency reduces intra-Sm DNA deletion. *Rad52*$^{+/+}$ and *Rad52*$^{–/–}$ B cells were stimulated with LPS plus IL-4 for 96 h. Sμ DNAs were amplified, cloned and sequenced. Each sequence is aligned with germline Sm sequence. Upstream and downstream germline Sm sequences involved in the intra-Sm recombination are at the top and bottom, respectively. The numbers on top and bottom of each aligned sequence indicate upstream and downstream breakpoints of recombined Sμ sequences. Microhomologies are bold and underlined. Sequences were derived from stimulated B cells of three pairs of *Rad52*$^{+/+}$ and *Rad52*$^{–/–}$ mice. Sequences containing microhomologies are in blue; point-mutations in red.

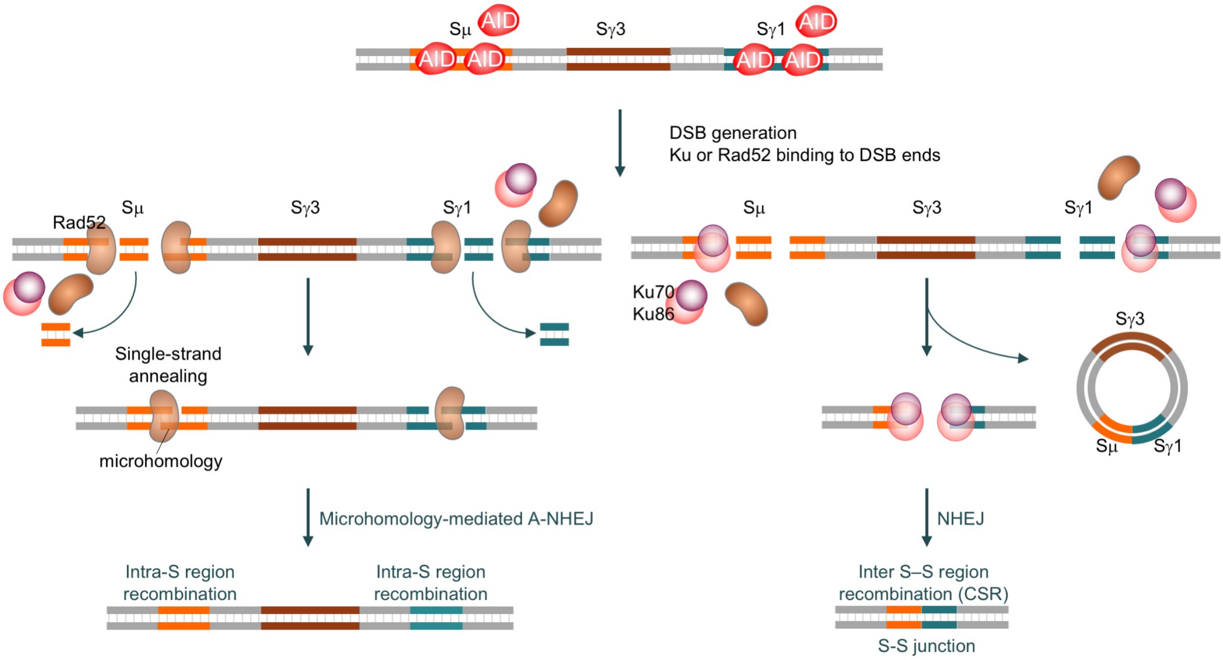

Supplementary Figure 5 | Rad52 competes with Ku70/Ku86 for binding to free ends of AID-mediated S region DSBs to modulate CSR. During CSR, multiple DSBs are introduced into an S region, some of these DSB ends are recombined, thereby generating intra-S region deletions. Ku70/Ku86, a core NHEJ factor, binds to DNA ends to repair DSBs by NHEJ, effectively leading to inter-S–S region recombination (CSR), which entails deletion of the intervening sequence between S regions as an extrachromosomal circle. Rad52, an HR element, binds preferentially to DNA single-strand overhangs and facilitates a (Ku-independent) microhomology-mediated A-NHEJ, which favors intra-S region recombination but can also mediate, particularly in the absence of the NHEJ pathway, inter-S–S region DSBs recombination.

Figure 1f

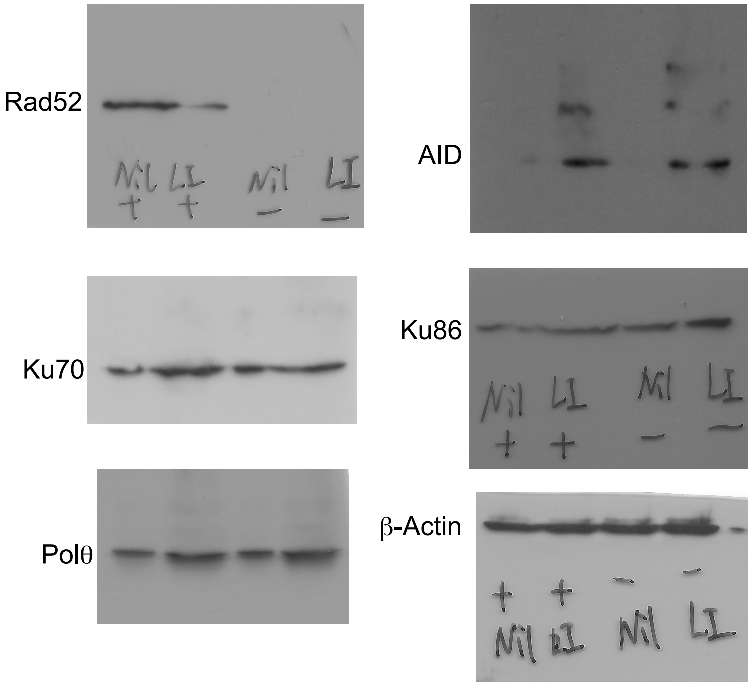

Figure 9a

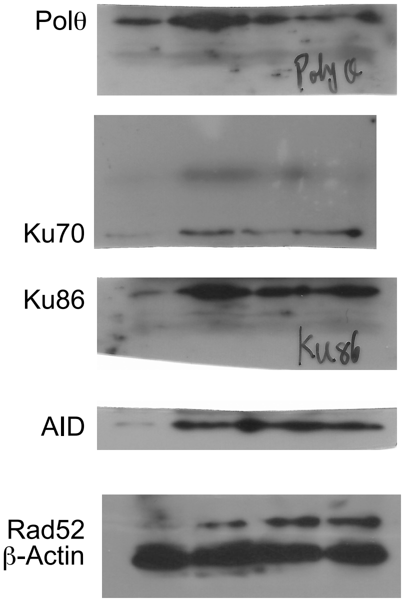

Supplementary Figure 6 | Uncropped scan images of important western blotting presented in Figure 1 and Figure 9.

Supplementary Table 1 | The phenotypes of Ku and Rad52 deficient B cells are inversely consistent.

	CSR	S-S junction Microhomology	Intra-Sμ region deletion	*c-Myc/IgH* translocation	Ref.
Normal (*Ku70*$^{+/+}$ *Ku86*$^{+/+}$*Rad52*$^{+/+}$)	++	++	++	++	
Ku70 or *Ku86* knockout	+	+++	+++	+++	1-5
Rad52 knockout	+++	+	+	+	This study

Supplementary References

1. Manis JP*, et al.* Ku70 is required for late B cell development and immunoglobulin heavy chain class switching. *J Exp Med* **187**, 2081-2089 (1998).
2. Casellas R*, et al.* Ku80 is required for immunoglobulin isotype switching. *EMBO J* **17**, 2404-2411 (1998).
3. Difilippantonio MJ*, et al.* DNA repair protein Ku80 suppresses chromosomal aberrations and malignant transformation. *Nature* **404**, 510-514 (2000).
4. Boboila C*, et al.* Alternative end-joining catalyzes robust *IgH* locus deletions and translocations in the combined absence of ligase 4 and Ku70. *Proc Natl Acad Sci USA* **107**, 3034-3039 (2010).
5. Boboila C*, et al.* Alternative end-joining catalyzes class switch recombination in the absence of both Ku70 and DNA ligase 4. *J Exp Med* **207**, 417-427 (2010).

Supplementary Table 2 | Primers for qRT-PCR, ChIP assays, retroviral construction, and detection of S-S junction, *c-Myc/IgH* translocation and intra-Sμ deletion.

	Forward primer	Reverse primer
Mouse genes		
Aicda	5'-AGAAAGTCACGCTGGAGACC-3'	5'-CTCCTCTTCACCACGTAGCA-3'
Rad52	5'-AGCCAGTATACAGCGGATGAA-3'	5'-GCCATGCGGCTGCTAATGTA-3'
Polθ	5'-TGGCTATATGGGCAGCACCT-3'	5'-CAGAGCAATGCCCTTGGATTT-3'
Ku70	5'-CACCAAGCGGTCTCTGACTT-3'	5'-AGAGAGGGCCTCAGGTAGTG-3'
Ku86	5'-AGGCCCAGGAAGCTCTATCA-3'	5'-GCACTCTTGGATTCCCCACA-3'
Gapdh	5′-TTCACCACCATGGAGAAGGC-3′	5′-GGCATGGACTGTGGTCATGA-3′
Cd79b	5′-CCACACTGGTGCTGTCTTCC-3′	5′-GGGCTTCCTTGGAAATTCAG-3′
Human genes		
AICDA	5'-GTCACCTGGTTCACCTCCTG-3'	5'-CTTGCGGTCCTCACAGAAGT-3'
RAD52	5'-GTAGGGAGAGGCTCTGGACA-3'	5'-GCAGGTGCTTAGGACCAAGT-3'
POLθ	5'-CTGCAGACAGAGGAATGCCA-3'	5'-TCTGCTGCAATGCTCCTGAA-3'
KU70	5'-GAAGCAAAAGGCCCAAGGTG-3'	5'-AGCAGCTCCTGCTTCTTCAG-3'
KU86	5'-GCAGTGTCACCTCTGTTGGA-3'	5'-GCTCGGATGCAGTCTATGCT-3'
CD79b	5'-AGGAACACGCTGAAGGATGG-3'	5'-CCACTTCACTTCCCCTGTCC-3'
Germline transcripts		
Iγ1-Cγ1	5′-TCGAGAAGCCTGAGGAATGTG -3′	5′-ATGGAGTTAGTTTGGGCAGCA-3′
Iγ3-Cγ3	5'-AACTACTGCTACCACCACCACCAG-3'	5'-ACCAAGGGATAGACAGATGGGG-3'
Post-recombination transcripts		
Iμ-Cγ1	5'-ACCTGGGAATGTATGGTTGTGGCTT-3'	5′-ATGGAGTTAGTTTGGGCAGCA-3′
Iμ-Cγ3	5'-ACCTGGGAATGTATGGTTGTGGCTT-3'	5'-AGCCAGGGACCAAGGGATAGAC-3'
Retroviral construction		
Mouse Rad52	5'-GCTAAACTGAGGTGATTACTCTGAGGTAAG-3'	5'-AATTCGAATTCAGTGACATCTTGAGTCTCA GGATGG-3'
ChIP assays		
Sμ	5'-GCTAAACTGAGGTGATTACTCTGAGGTAAG-3'	5'-GTTTAGCTTAGCGGCCCAGCTCATTCCAGT-3'
Sγ1	5'-ATAAGTAGTAGTTGGGGATTC-3'	5'-CTCAGCCTGGTACCTTATACA-3'
Sγ3	5'-AATCTACAGAGAGCCAGGTGG-3'	5'-TGGTTTTCCATGTTCCCACTT-3'
Cμ	5'-CAGCACCATTTCCTTCACCTGGAACTACCA-3'	5'-GGCTAGGTACTTGCCCCCTGTCCTCAGTGT-3'
S-S junction		
Sμ-Sγ1		
First round	5'-AACTCTCCAGCCACAGTAATGACC-3'	5'-CTGTAACCTACCCAGGAGACC-3'
Second round	5'-GAGAAGGCCAGACTCATAAAGCT-3'	5'-GTCGAATTCCCCCATCCTGTCACCTATA-3'
Sμ-Sα		
First round	5'-AACTCTCCAGCCACAGTAATGACC-3'	5'-TCCAGCAAAGCTCAGGCTAGAAC-3'
Second round	5'-GAGAAGGCCAGACTCATAAAGCT-3'	5'-AGTCCAGTCATGCTAATTCACC-3'
c-Myc/IgH translocation		
First round	5'-TGAGGACCAGAGAGGGATAAAAGAGAA-3'	5'-CAGTCTCCGGCTATCACAAGCCT-3'
Second round	5'-CACCCTGCTATTTCCTTGTTGCTAC-3'	5'-GACACCTCCCTTCTACACTCTAAACCG-3'
Intra-Sμ deletion		
First round	5′-AACTCTCCAGCCACAGTAATGACC-3′	5′-AGGGTAGGAGGAAGGTGGGTTATG-3′
Second round	5'-GAGAAGGCCAGACTCATAAAGCT-3'	5'-TCCACTGTAGCACACAGTCT-3'
